# Kallikrein-Related Peptidase 6 Contributes to Murine Intestinal Tumorigenesis Driven by a Mutant *Adenomatous polyposis coli* Gene

**DOI:** 10.3390/cancers16223842

**Published:** 2024-11-15

**Authors:** Teodora G. Georgieva, Dalila Darmoul, Hwudaurw Chen, Haiyan Cui, Photini F. S. Rice, Jennifer K. Barton, David G. Besselsen, Natalia A. Ignatenko

**Affiliations:** 1 Genetically Engineered Mouse Models Core, The University of Arizona Bio5 Institute, Tucson, AZ 85721-0240, USA; teodorag@arizona.edu; 2 Department of Cellular and Molecular Medicine, The University of Arizona Cancer Center, Tucson, AZ 85724-5024, USA; hwu@arizona.edu (H.C.); hcui@uacc.arizona.edu (H.C.); 3 Institut de Biologie Paris-Seine, Sorbonne Université, UMR CNRS 8256, INSERM ERL U1164, Biological Adaptation and Ageing, 75005 Paris, France; dalila.darmoul@inserm.fr; 4 Department of Biomedical Engineering, The University of Arizona, Tucson, AZ 85721-0240, USA; psrice@arizona.edu (P.F.S.R.); barton@arizona.edu (J.K.B.); 5 University Animal Care, The University of Arizona, Tucson, AZ 85721-0101, USA; besselsd@arizona.edu; 6 Department of Cellular and Molecular Medicine, The University of Arizona, Tucson, AZ 85724-5024, USA

**Keywords:** kallikrein-related peptidase 6, KLK6 conditional knockout mouse, *APC* mutation, β-catenin, transforming growth factor β2 isoform, ERK1/2 mitogen-activated protein kinase

## Abstract

Background/Objectives: The objective of this study was to assess the role of a secreted serine protease, kallikrein-related peptidase 6 (KLK6), during colorectal tumorigenesis driven by a mutant *Adenomatous polyposis coli (APC)* tumor suppressor gene. A first analysis of KLK6 expression in the intestinal tract of *Apc*-mutant multiple intestinal neoplasia (*Apc^Min/+^*) mice revealed up to four-fold induction of *Klk6* mRNA levels in adenomas relative to its level in the adjacent mucosa. Methods and Results: The presence of KLK6 protein in the adenomatous areas was confirmed by immunohistochemistry and optical coherence tomography/laser-induced fluorescence (OCT/LIF) imaging. To assess the contribution of the KLK6 expression on the *Apc*-mutant intestinal and colon tumorigenesis, we engineered a mouse with floxed alleles of the *Klk6* gene (*Klk6^lox/lox^*) and crossed it with a mouse expressing the truncated APC protein under control of the intestinal tract-specific human *CDX2P9.5-NLS Cre* transgene (*CPC;Apc^fl/fl^*;*Klk6^+/+^*). We found that *CPC;Apc^fl/fl^* mice with disrupted *Klk6* gene expression (*CPC;Apc^fl/fl^*;*Klk6^fl/fl^*) had a significantly smaller average size of the small intestinal and colon crypts (*p* < 0.001 and *p* = 0.04, respectively) and developed a significantly fewer adenomas (*p* = 0.01). Moreover, a decrease in high-grade adenomas (*p* = 0.03) and adenomas with a diameter above 2 mm (*p* < 0.0001) was noted in *CPC;Apc^fl/fl^*;*Klk6^fl/fl^* mice. Further molecular analysis showed that *Klk6* gene inactivation in the small intestine and colon tissues of *CPC;Apc^fl/fl^*;*Klk6^fl/fl^* mice resulted in a significant suppression of transforming growth factor β2 (TGF-β2) protein (*p* ≤ 0.02) and mitogen-activated protein kinase (MAPK) phosphorylation (*p* ≤ 0.01). Conclusions: These findings demonstrate the oncogenic role of KLK6 in the mutant *Apc*-mediated intestinal tumorigenesis and suggest the utility of KLK6 for early diagnosis of colorectal tumors.

## Simple Summary

Mutations in the *Adenomatous polyposis coli* (APC) tumor suppressor gene are common in colorectal cancer. We found that kallikrein 6 (KLK6), a secreted serine protease, is overexpressed in the intestinal tract of *Apc*-mutant multiple intestinal neoplasia (*Apc^Min/+^*) mice and can be detected in mouse colon adenomas using imaging techniques with fluorescence-labeled KLK6 antibody. We generated a novel mouse model of the *Apc*-mutant tumorigenesis with an intestine-specific knockout of the *Klk6* gene. *Klk6* gene disruption in this model suppressed the number, size, and histological grade of the intestinal adenomas via a decrease in TGF-β expression and mitogen-activated protein kinase phosphorylation. Our findings imply that KLK6 overexpression accelerates *Apc*-mutant tumorigenesis and can be utilized for the early detection of colon adenomas.

## 1. Introduction

Colorectal cancer (CRC) continues to be the third most common cancer in both men and women [1]. CRC develops upon acquisition of a series of cancer driver mutations and genomic alterations in the tumor suppressor genes and protooncogenes, which are involved in multiple signaling pathways, such as Wnt, RAS-MAPK, TGF-β, and DNA mismatch-repair [2]. The biological and molecular characteristics of CRC tumors are complex and often cause difficulties in CRC detection and treatment. The mutations in the Adenomatous Polyposis (*APC*) tumor suppressor gene are prevalent in colorectal tumorigenesis and lead to the activation of the canonical Wnt signaling network [3]. Somatic *APC* mutations in the intestinal epithelium drive neoplastic transformation through the chromosomal instability (CIN) molecular pathway, which results in the development of sporadic colon tumors [2,4]. Germ-line APC mutations cause the autosomal dominantly inherited syndrome Familial Adenomatous Polyposis (FAP), with multiple adenomas developing in the gastrointestinal tract [5]. Genomic analysis of APC-driven adenomas and adjacent mucosa of FAP patients revealed that the tissue adjacent to the adenoma site carries 23% of the somatic mutations and genomic variations in known cancer driver genes, which are often detected in adenomas (i.e., *APC*, *K-RAS*, *FBXW7*, *TCFL2*) [6].

The elevated expression and activity of proteases and their contribution to cell invasion is a well-documented hallmark of cancer [7,8,9]. As proteolytic enzymes, kallikrein-related peptidases can interact with different signaling pathways inside and outside of the tumor, thus playing an important role in the regulation of the tumor microenvironment [10].

Kallikrein-related peptidase 6 (KLK6) belongs to the family of 15 kallikrein peptidases. KLK6 is involved in various physiological and pathological processes, such as immunity [11,12] and skin desquamation [13]. KLK6 overexpression has been reported in various human cancers, including glioma, ovarian, gastric, skin, urinary bladder, and salivary gland tumors [14,15]. In CRC patients, KLK6 mRNA levels correlated with increased serosal invasion, liver metastasis, advanced Duke’s stage, and poor disease prognosis [16,17,18,19]. The high *KLK6* mRNA level was reported in non-malignant adenomatous tissue of CRC patients when compared to the KLK6 mRNA levels in the adjacent normal mucosa and malignant tumors [20].

KLK6, like other members of the serine protease family, is synthesized as a prepropeptide, with the signal peptide removed in the rough endoplasmic reticulum, generating an inactive zymogen form (proKLK6). KLK6 activity outside of the cell is controlled by other proteases, such as plasmin and KLK5, and negative feedback inhibition via autolytic inactivation or by serine protease inhibitors (serpins, α2-antiplasmin, antithrombin, α1-antichymotrypsin) [21,22,23]. Activated KLK6 enzyme hydrolyzes extracellular matrix proteins, such as collagen, fibronectin, laminin, and fibrinogen, thus facilitating cancer cell invasion [24].

The analysis of the kallikrein-related peptidase family in CRC tumor samples from The Cancer Genome Atlas (TCGA) database identified a distinct pattern of overexpression of KLK6 along with elevated expression of KLK7, KLK8, and KLK10 [18]. RNA-Sequencing data analysis of kallikrein genes across 15 different cancers from the TCGA database highlighted *KLK6*, *KLK7*, *KLK8*, and *KLK10* as the candidate genes for further evaluation as colon cancer biomarkers [25]. Specifically, KLK6 was validated as a suitable prognostic predictor for colon adenocarcinoma progression [26]. It was found that 75% of the CRC tumors with KLK6 overexpression carried *APC* mutations and 69% had K-RAS mutations, which indicates that KLK6 contributes to colorectal tumorigenesis downstream of the major colon tumorigenesis pathways [18,27].

Investigations into the KLK6 protumorigenic role in the K-RAS-mutated CRC tumors showed that KLK6 regulates cell proliferation, epithelial–mesenchymal transition (EMT), and invasion via a specific mRNA-micro RNA (miRNA) gene-interaction network [28]. KLK6 stimulates the TGF-β signaling pathway and correlates with the expression of the RNA-binding protein LIN28B and HMGA2 (high-mobility group AT-hook 2) transcription factor, which are both essential regulators of cell invasion and metastasis [29]. KLK6 can also enhance cancer cell proliferation through the cleavage and activation of protease-activated receptor 2 (PAR-2) [19,30]. Additionally, KLK6 plays a role in the tumor microenvironment by stimulating production of tumor necrosis factor alpha (TNF-α) through the activation of protease-activated receptor 1 (PAR-1) [31].

The development of animal models has been crucial for studying colorectal tumorigenesis. Particularly, the *Apc^Min/+^* (Multiple Intestinal Neoplasia) mouse model has provided valuable insights into the pathogenesis of CRC, the role of APC in tumor suppression, and the underlying mechanisms of polyp formation. The *Apc^Min/+^* mouse carries a dominant truncating mutation at codon 850 of the *Apc* gene, which leads to the formation of numerous polyps in the mouse intestinal tract that resemble human disease [32].

To enhance our understanding of KLK6’s role in mutant *APC*-driven CRC tumor development, we analyzed KLK6 expression in the intestinal tract of the *Apc^Min/+^* mouse. We also developed and characterized a genetically engineered mouse (GEM) model with conditional inactivation of both *Apc* and *Klk6* genes in the intestinal epithelium. We found that the disruption of the *Klk6* gene in this mouse model leads to a suppression of mutant *Apc*-mediated tumorigenesis.

## 2. Materials and Methods

### 2.1. Animals

*Apc^Min/+^* mice. C57BL/6J- *Apc^Min/+^* (Multiple Intestinal Neoplasia) mice were obtained from the Jackson Laboratory (Bar Harbor, ME, USA) https://www.jax.org/strain/002020 (accessed on 12 November 2024). These mice have a dominant mutation at codon 850 of the murine *Apc* gene that converts a leucine into a stop codon and causes the truncation of the APC protein [33].

*CDX2P9.5-NLS Cre (CPC)* mice. The *CPC* mice were obtained from the Jackson laboratory. https://www.jax.org/strain/009350 (accessed on 12 November 2024). In the *CPC* transgenic mouse model, Cre recombinase expression is driven by a 9.5 kb human caudal type homeobox 2 (*CDX2*) promoter/enhancer sequence and is localized preferentially in the small intestinal and cecal epithelium. Cre recombinase expression is present during late gestation and in adult tissues from the crypt base to the luminal surface [34].

*Apc* conditional knockout (*Apc^lox/lox^*) mice. The C57BL6/J-*Apc^loxP/loxP^* strain carries conditional alleles of the *Apc* gene with LoxP sites flanking exon 14 (Strain number 01XAA, Mouse Repository, NCI-Frederick). https://frederick.cancer.gov/resources/repositories/nci-mouse-repository (accessed on 12 Noember 2024).

*Klk6* conditional knockout *(Klk6^lox/lox^)* mice. We generated a mouse model with a conditional disruption of the *Klk6* gene by inserting *loxP* sites at the flanks of exon 5, which encodes for the aspartic acid (D) residue of the catalytic triad sequence of *Klk6* (*Mus musculus Klk6*, NP_035307.1, located on Chr. 7, NC_000073.6 (43824438.43832027)), as described in detail in the Supplementary Materials, Generation of Klk6 Conditional Knockout Mouse Model.

All mouse strains were bred in the University of Arizona’s Animal Care Facility, with approval by The University of Arizona Institutional Animal Care and Use Committee (IACUC). *Apc^Min/+^* animals were obtained by crossing *Apc^Min/+^* males with C57BL/6J females. The *CPC;Apc^fl/fl^* mice with a functional *Klk6* gene (*CPC;Apc^fl/fl^;Klk6^+/+^*) were generated by breeding *CDX2P9.5-NLS Cre (CPC)* mice with *Apc^lox/lox^* and *Klk6^lox/lox^* mice. Genotyping of the *Apc^Min/+^*, *Apc ^lox/lox^*, *Klk6 ^lox/lox^*, and *CPC*, and their cross strains was performed by Transnetyx Inc. (Cordova, TN) based on the protocols from the Jackson laboratory (*Apc^Min/+^*, CPC strains), the NCI-Frederick Mouse Repository (*Apc^lox/lox^ strain*), and the designed Polymerase Chain Reaction (PCR) protocol for the *Klk6^lox/lox^* strain (Supplementary Materials, Mouse Klk6 Gene Conditional Knockout Primers). Animals were housed in groups of one to five in individually ventilated microisolator cages under fluorescent lighting on a 14:10 light–dark cycle. Irradiated feed (Teklad Global diet 2919, Envigo RMS Division, Indianapolis, IN, USA) and hyperchlorinated reverse osmosis drinking water were available ad libitum for the duration of the experiment.

### 2.2. Tissue Collection, Histological Analysis, and Tumor Scoring

Mice of different genotypes were euthanized by CO_2_ inhalation. The entire small intestine and colon were removed, flushed with phosphate-buffered saline (PBS) solution, opened longitudinally, and laid flat, mucosal surface up. After excision, small intestines and colons were placed on filter paper, cut open longitudinally, and flattened to fully expose the intestinal mucosa. The tissues were examined visually and photographed. Tissue samples were well-oriented with longitudinally cut crypts to precisely assess the overall intestinal tissue architecture. The proximal, middle, and distal small intestines and the colons of the 6-month-old *CPC;Apc^fl/fl^;Klk6^+/+^*, *CPC;Apc^fl/fl^;Klk6^+/fl^*, and *CPC;Apc^fl/fl^;Klk6^fl/fl^* mice were harvested for histological analysis and the occurrence of tumors.

The small intestines and colons were fixed in 10% buffered formalin or Histochoice (Sigma-Adrich, Inc., St. Louis, MO, USA) for 24 h, then washed with cold Phosphate Buffered Saline (PBS) and stored in 70% ethanol at 4 °C until processed, paraffin-embedded, and cut longitudinally into 6 μm for hematoxylin and eosin (H&E) staining or 3 μm for immunohistochemistry (IHC) sections every 250 µm. The H&E slides were coded to blind the pathologist to the strain of the animals. Histological features of the tumors from the entire small intestine and colon were evaluated by a board-certified veterinary pathologist. The grade of dysplasia for each adenoma examined histologically was determined based on criteria outlined in a review of the pathology of mouse models of intestinal cancer [35]. The intestines and colons from each mouse were also scored on the severity of mucosal epithelial changes, degree of inflammation, and extent of pathology, based on the lesion scoring system for mouse intestinal lesions [36]. Tumors in the proximal, middle, and distal portions of the small intestines and the colons were counted in mice of different genotypes (9–12 mice per genotype) with an LSGA epi-illuminator dissecting microscope (Olympus Optical Co., Ltd., Tokyo, Japan) at 20× magnification. Tumor size was determined by measuring the maximum diameter of each discrete tumor, and tumors were counted when they had a minimum diameter of 0.5 mm.

Photo micrographs were obtained using a brightfield microscope (BX41, Olympus, Japan) and a 12.5-million-pixel resolution camera system (DP71 with Microsuite Analytical Suite V.5, Olympus).

### 2.3. Crypt Scoring

The H&E-stained histological sections of the distal small intestine and distal colon from *CPC;Apc^fl/fl^;Klk6^+/+^*, and *CPC;Apc^fl/fl^;Klk6^fl/fl^* mice were evaluated using standard light microscopy. The crypt measurement was performed on the IHC sections stained for K-67. The slides were scanned with a slide scanner Lamina3D HISTECH. LTD (Perkin Elmer, Villebon sur Yvette, France) and were analyzed using INFORM software (Perkin Elmer, Villebon sur Yvette, France). Morphometric parameters were assessed using a Case Viewer software V2.4 (Perkin Elmer, Villebon sur Yvette, France). Only crypts with apparently complete, full-sized intestinal villi that were not exhibiting bending or mechanical damage were measured. Measurements were performed using photomicrographs taken at 20× magnification. Crypt depth (in μm) was measured from the villus border to the bottom of the crypt, and fifteen measurements of full-length crypts were obtained from each sample.

The choice of 15 measurements for crypt length in histology is based on ensuring statistical reliability and validity. Measurements were taken from at least three fields of view to ensure that the data were not biased by local variations. The average length of the small intestinal and colon crypts in the *CPC;Apc^fl/fl^;Klk6^+/+^* (2 animals, 30 crypts per location) and *CPC;Apc^fl/fl^;Klk6^fl/fl^* (4 animals, 60 crypts per location) genotypes were compared.

### 2.4. Quantitative Reverse-Transcription Polymerase Chain Reaction (qRT-PCR) Analysis

Total RNA from the mouse intestinal tract was isolated using the Qiagen RNeasy Kit (Cat. # 74136, Qiagen GmbH, Hilden, Germany) according to the manufacturer’s protocol. Portions of the ileum, colon, liver, spleen, and kidney were used for RNA isolation from 2-month-old wild-type and CPC;*Klk6^loxP/loxP^* mice. Portions of the ileum and colon were used for RNA isolation from 15-week-old *Apc^Min/+^* mice and 6-month-old *CPC;Apc^fl/fl^;Klk6^+/+^*and *CPC;Apc^fl/fl^;Klk6^fl/fl^* mice. The tissues (~30 mg) were homogenized in 600 μL of buffer RLT using a Polytron homogenizer followed by centrifugation. Reverse transcription was completed using the Applied Biosystems High-Capacity cDNA Reverse Transcription Kit (Part #4368814, Applied Biosystems, Foster City, CA, USA). Total RNA (0.2 μg) was transcribed into cDNA in a 20 μL reaction using random hexamers under the thermal conditions recommended by the protocol. Quantitative *Klk6* mRNA RT-PCR was performed using TaqMan^®^ gene expression assay (Applied Biosystems, Thermo Fisher Scientific, Inc.), which covers the exon boundary 5–6, of the mouse *Klk6* mRNA RefSeq (Accession number: NM_011177.2, AssayID: Mm00478322_m1). This region is shared by all isoforms of the mouse *Klk6*. The qPCR analysis of mouse ornithine decarboxylase 1 (*Odc1*) mRNA was done using the TaqMan^®^ gene expression assay for mouse *Odc1* (AssayID: Mm02019269_g1). Real-time PCR amplification was performed with the ABI PRISM 7700 SDS instrument (Applied Biosystems, Foster City, CA, USA), using the universal thermal cycling conditions recommended by the Assay-on-Demand products protocol. No template controls were included on each plate to monitor for potential PCR contamination. Each reaction was run in triplicate. To determine the expression levels of *Klk6* and *Odc1* genes in mice of the different genotypes, the C_T_ values of genes of interest in wild-type (wt) and knockout mice were normalized by the endogenous reference, mouse β2-microglobin gene (β2M, FAM (Hs99999907_m1), (ΔC_T_ = C_T(Klk6 or Odc1)_ − C_T(β2M)_). The normalized individual expression of genes in mice of different genotypes was calculated as 2^−ΔCT^ and presented as the mean ± SD expression for each group of animals. The comparative Ct method was used to determine a relative expression of *Klk6* in tumors versus adjacent tissue. The Ct values of *Klk6* in the normal and tumor tissues were normalized by the endogenous reference, and normalized C_T_ values from tumors in each animal were compared with Ct values from normal tissue in the same animal (ΔΔC_T_ = ΔC_T(treatment)_ − ΔC_T(control)_). The relative expression was calculated as 2^−ΔΔCT^.

### 2.5. Immunohistochemistry

Staining was performed on 3 μm sections of ileum and colon tissue cut from formalin-fixed, paraffin-embedded (FFPE) blocks. IHC was performed on a Leica BOND RXm autostainer (Leica Microsystems Inc., Deer Park, IL, USA) according to the manufacturer’s instructions. For KLK6 IHC, the human-specific KLK6 antibody (R&D, AF2008, immunogen Gly17-Lys244, Accession number Q92876, dilution 1:150) was used in the absence of a commercially available mouse-specific primary KLK6 antibody. The secondary biotinylated anti-goat IgG antibody (H&L Vector Laboratories Inc., Burlingame, CA, USA, dilution 1:200) was used for detection of the KLK6 primary antibody.

Indeed, KLK6 human and mouse protein sequence alignment in the Supplementary Materials, KLK6 Human–Mouse Protein Alignment, shows highly conservative sequences between human and mouse KLK6 orthologs. The mouse KLK6 contains two additional amino acids at the C-terminus. Staining with normal goat IgG control antibody (R&D Systems, Minneapolis, MN, USA, Cat#AB-108-C, dilution 1:150) was done to evaluate the specificity of the human KLK6 antibody in the mouse microscopically normal colon (Figure S1A) and adenoma (Figure S1B) tissue samples.

The other primary antibodies used were Ki-67 (Abcam, Cambridge, UK, Cat. #AB15580, rabbit polyclonal, dilution 1:500), β-catenin (Cell Signaling Technology, Inc., Danvers, MA, USA, Cat. #9562, rabbit polyclonal, dilution 1:300), and phospho-ERK1/2 (p-ERK1/2) (Cell Signaling Technology Inc., Cat.# 4370, rabbit monoclonal, dilution 1:200). Detection of these primary antibodies was accomplished using the Bond Polymer Refine Detection system (Leica cat#DS9800). The IHC staining was performed using the tissue of animals from *CPC;Apc^fl/fl^;Klk6^+/+^*and *CPC;Apc^fl/fl^;Klk6^fl/fl^* genotypes, and three animals per genotype were analyzed. Staining intensity for β-catenin was scored as previously described according to the following system: 0—no staining; 1—weak diffuse staining (may contain strong intensity in <10% of the cells); 2—moderate staining in 10% to 90% of the cells; 3—more than 90% of the cells stained with strong intensity. Scores were based on the sum of the intensity of staining multiplied by the percent of stained tissue area. Staining for all endpoints was analyzed in at least three fields per slide. The slides stained for Ki-67 were scanned using a slide scanner Lamina3D HISTECH. LTD (Perkin Elmer, Villebon sur Yvette, France), and the resulting images were preparedfor further evaluation with Case Viewer V2.4 software (To determine the percentage of Ki-67 positive cells, four selected images of each slide from tissue samples of *CPC;Apc^fl/fl^;Klk6^+/+^*and *CPC;Apc^fl/fl^;Klk6^fl/fl^* genotypes were captured and analyzed for positive staining using iINFORM Tissue Analysis.software V2.6.0 (Perkin Elmer, Villebon sur Yvette, France).

### 2.6. Western Blot Analysis

Portions of the distal small intestine and distal colon were homogenized on ice in radioimmunoprecipitation assay (RIPA) lysis buffer (PBS, 1% NP-40, 0.5% sodium deoxycholate, 0.1% SDS) containing Halt™ Protease&Phosphatase Inhibitor Cocktail (ThermoScientific, Cat#1861280). Homogenized samples were incubated on ice for 30 min with occasional shaking and centrifuged at 10,000× *g* for 15 min at 4 °C centrifuge. The supernatants were transferred to new minifuge tubes, and total protein levels in supernatants were measured using a Bio-Rad DC protein assay kit (DC™ Protein Assay Kit I, Cat. #5000111, Life Science, Bio-Rad Laboratories, Inc., Hercules, CA, USA). Equal amounts (50 μg) of the protein were separated on Mini-PROTEAN TGX Bio-Rad Precast Gels Any-kD (Criterion TGX Stain-free, Cat#5678123; Bio-Rad Laboratories, Inc. Hercules, CA, USA) and transferred to Nitrocellulose membrane (Cat#160094, Bio-Rad Laboratories, Inc.) for 30 min. The membranes were processed for Western blot analysis as previously described [28]. The primary and secondary antibodies used in this study and the blotting conditions are presented in Table S1. Proteins of interest were detected with an enhanced chemiluminescent detection (ECL) reagent (Super Signal West Pico or West Femto Chemiluminescent Substrate, Cat#34077 or #34095, respectively, ThermoFisher Scientific, Waltham, MA, USA) and exposed to film (Autoradiography film, Cat#30-810L, Genesee Scientific Co., El Cajon, CA, USA). All western blots were repeated at least twice. An image processing program (Image J, version 1.53) was used to quantify the intensity of Western blot bands where specified. Protein bands of interest were normalized to β-actin expression level.

### 2.7. Imaging of Mice Using Fluorescence Dye-Labeled KLK6 Antibody

The human KLK6 antibody (Catalog#AF2008, R&D Systems, Inc., Minneapolis, MN, USA) was labeled with Alexa Fluor 555 fluorescent dye (peak absorption 555 nm, peak emission 565 nm) using an APEX™ Antibody Labeling Kit according to the manufacturer’s protocol (MP10468, Molecular probes^®^, Eugene, OR, USA). Two-hundred µg of KLK6 antibody was incubated with fluorescence dye overnight, and the labeled KLK6 antibody at a final concentration of 20 µg/mL was stored at −20 °C. The labeled antibody was diluted 1:50 in PBS) for detection of KLK6 in animal tissue.

The imaging of *Apc^Min/+^* mouse colon tissue was performed using the endoscopic ultra-high resolution optical coherence tomography/laser-induced fluorescence (OCT/LIF) system as previously described [37]. Briefly, the system consists of a 2 mm diameter endoscope with a quartz distal sheath, which is inserted approximately 35 mm into the distal colon. The internal optics can translate and rotate to general longitudinal images of the colon. The OCT system utilizes an 890 nm center wavelength broadband source and generates cross-sectional images with an approximate resolution of 3.5 µm axially and 15 µm laterally. The LIF system included a 543 nm He–Ne laser, which expanded to an approximately 1 mm diameter spot on the colon tissue. Remitted light was captured, filtered to exclude the laser source light, and expanded onto a spectrometer to measure the fluorescence emission spectrum.

Four 15-week-old *Apc^Min/+^* and four age-matched *Apc* wild-type mice were placed into empty micro-isolator cages with wire bottom inserts to prevent coprophagia and kept without food for 16–18 h prior to imaging to clear the intestinal tract. Mice had free access to non-flavored pediatric electrolyte replacement supplement solution during fasting. Mice were anesthetized with a mixture of 100 mg/kg ketamine–10 mg/kg xylazine or 2.5% Avertin administered intraperitoneally prior to imaging. The distal colon was flashed with 0.9% sodium chloride irrigation solution, United States Pharmacopeia (USP), to clear residual fecal material. Following colonic lavage, mice were placed on a warming pad, and the colon endoscopy was performed with the OCT/LIF system. Immediately after OCT/LIF imaging, mice were sacrificed by CO_2_ inhalation. The colons were removed, and the solution with labeled KLK6 antibody was applied to the tissues. Ten minutes later, ex vivo tissues were imaged using the OCT/LIF system.

### 2.8. Statistical Analysis

The data are presented as a mean value per genotype and the standard deviation.

The ANOVA non-parametric Kruskal–Wallis test was used to analyze mRNA expression between genotypes and the intensity of immunohistochemistry staining. A *p*-value < 0.05 was considered statistically significant. The statistical analysis of crypt length was performed using the GraphPad Prism software version 9.0 (GraphPad Software Inc., La Jolla, CA, USA). The difference in crypt length between *CPC;Apc^fl/fl^;Klk6^+/+^* and *CPC;Apc^fl/fl^;Klk6^fl/fl^* mice was assessed using a non-parametric Mann–Whitney test, and *p* < 0.05 was considered significant.

We used Poisson regression to compare the total number of adenomas and histopathology grade between genotypes. To analyze adenoma size, we calculated the percentage of adenomas in different size ranges (0–5 mm, 0.5–1 mm, 1–2 mm, 2–3 mm, and >3 mm) for each genotype and compared them using the chi-square test. We also performed paired comparisons for all measurements and adjusted the *p*-values using Bonferroni multiple comparisons. Additionally, we utilized the ANOVA single-factor test to assess the differences in protein expression levels between genotypes, and *p* ≤ 0.5 was considered significant.

## 3. Results

### 3.1. Analysis of Klk6 Expression in Apc^Min/+^ Mice

**Figure 1:**
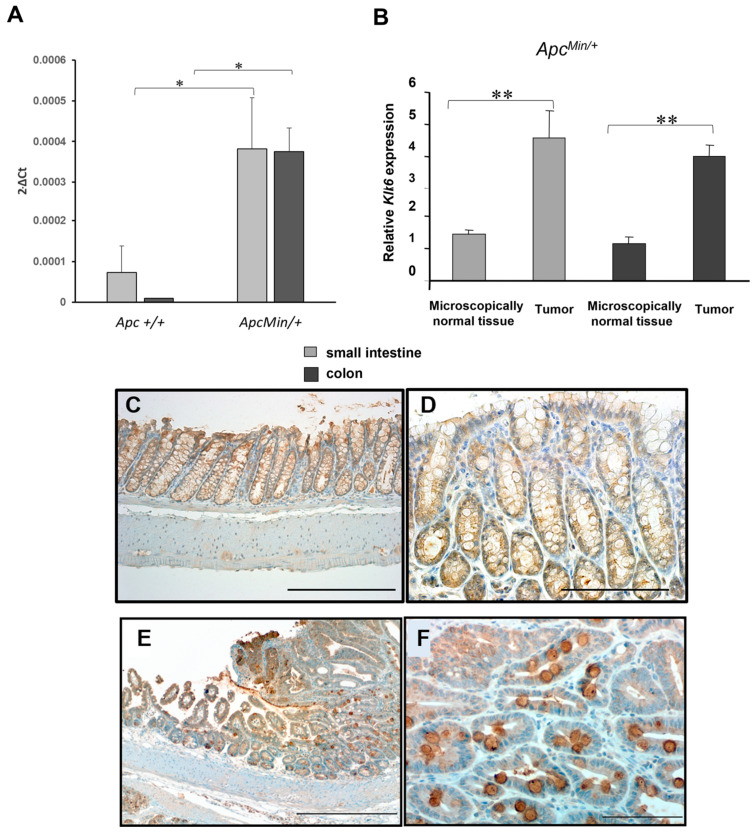
KLK6 expression in Apc^Min/+^ mice. (**A**) QRT-PCR analysis of Klk6 transcript levels in the tumor-free intestinal and colonic tissues of 15-week-old Apc^Min/+^ mice (n = 6) and 15-week-old Apc wild-type mice (n = 3). Data are presented as the normalized average Klk6 gene expression per mouse, 2^−ΔCt^. (**B**) Klk6 transcript levels in the small intestinal and colon adenomas of Apc^Min/+^ mice versus the adjacent microscopically normal tissues by qRT-PCR (n = 3 per group). Data are presented as the relative Klk6 expression, where the Klk6 level was normalized by the endogenous reference and compared to a calibrator (wild-type RNA) (2^−ΔΔCt^). * *p* = 0.04, ** *p* < 0.03 by Kruskal–Wallis test. (**C**,**D**) KLK6 IHC staining in microscopically normal colonocytes of 15-week-old Apc^Min/+^ mouse. Scale bar: 200 μm (**C**), 100 μm (**D**). (**E**) Representative pattern of KLK6 expression in the colonic adenoma of the Apc^Min/+^ mouse by IHC staining. Scale bar: 500 μm. (**F**) KLK6 protein localization in the goblet cells within the dysplastic epithelium. Scale bar: 100 μm.

We measured *Klk6* mRNA levels in microscopically normal tissue from the small intestine and colon of 15-week-old Apc^Min/+^ mice using qRT-PCR. As shown in Figure 1, we observed a more than three-fold increase in the level of Klk6 transcript in the Apc^Min/+^ mice as compared to the *Apc* wild-type (*Apc^+/+^*) mice (Figure 1A, *p* = 0.04). In the small intestinal and colon adenomas of *Apc^Min/+^* mice, *Klk6* transcript levels were nearly four-fold higher than in adjacent microscopically normal tissue (Figure 1B, *p* < 0.03). KLK6 IHC staining was done in the colon of a 15-week-old *Apc^Min/+^* mouse using a human-specific KLK6 antibody. As shown in Figure 1, the representative image of KLK6 IHC staining in the microscopically normal colonic epithelium of an Apc^Min/+^ mouse demonstrates low staining for KLK6 in the goblet cells at low and high magnifications (Figure 1C and Figure 1D, respectively). The adenoma tissue was stained strongly for KLK6 in the cytoplasm and luminal surface areas, and the less intense staining for KLK6 is seen in the adjacent microscopically normal tissue (Figure 1E,F). These data demonstrate that KLK6 expression is elevated in the adenomatous tissue of *Apc^Min/+^* mice.

### 3.2. Detection of KLK6 in Mouse Colon Using Fluorophore-Labeled Antibodies

**Figure 2:**
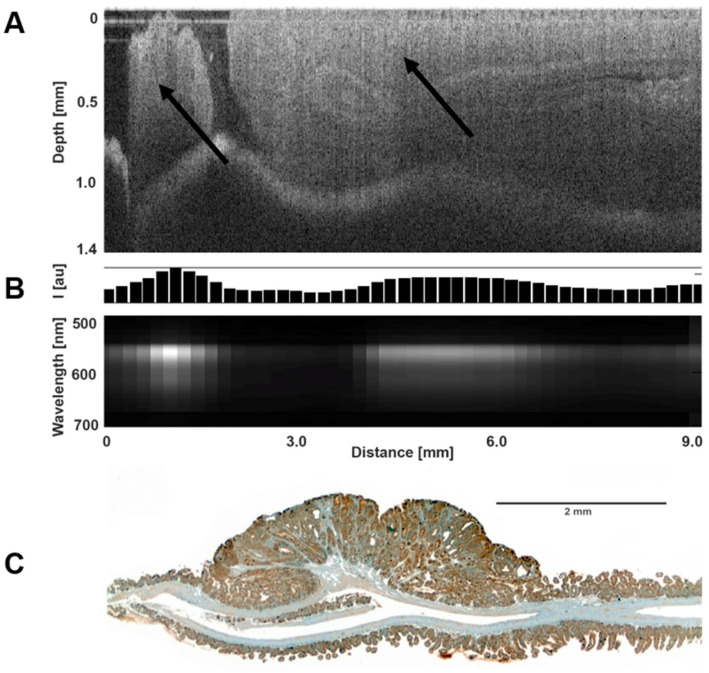
Detection of KLK6 in the mouse colon using KLK6-targeted Alexa Fluor 555 conjugated antibody. (**A**) Representative OCT image. Arrows indicate the large adenomas in the colon of the *Apc^Min/+^* mouse. (**B**) LIF image. Strong fluorescence is seen in the regions of the large adenomas. Bar chart shows the intensity of the fluorescence emission at 565 nm. (**C**) Immunostaining analysis of KLK6 protein expression in colonic adenoma and surrounding tissue in the *Apc^Min/+^* mouse.

Since we showed above that KLK6 is overexpressed in the adenomas of *Apc^Min/+^* mice, and because it is a secreted protein, we attempted to visualize KLK6 in the mouse colon using fluorescent imaging with Alexa Fluor-conjugated anti-KLK6 antibody. The 15-week-old *Apc^Min/+^* mice (n = 4) and *Apc^+/+^* littermates (n = 4) were imaged by the dual-modality OCT/LIF endoscopic system, as described in the Materials and Methods section. The OCT system generates high-resolution images of colon tissue morphology, which allows distinguishing normal and adenomatous areas (Figure 2A). Following mouse sacrifice, the colon tissue was exposed to KLK6-Alexa Fluor 555 conjugated antibody, and tissue imaging was performed with the LIF system. A strong KLK6-specific fluorescence signal was detected in the adenoma regions of the *Apc^Min/+^* mice (Figure 2B), while the control mice showed no appreciable fluorescence in any of the tissues imaged.The presence of KLK6 protein in adenomas was also confirmed by immunohistochemistry on resected colons (Figure 2C). These data demonstrate for the first time that KLK6 can be visualized in the adenomatous tissue using in vivo imaging with an antibody-specific fluorescent contrast agent. This experiment suggests that KLK6 may be utilized as a potential marker of adenomas.

### 3.3. Conditional Inactivation of Klk6 in the Mouse Intestinal Tract

To understand the function of KLK6 protein in intestinal and colon tumorigenesis, we utilized a mouse model with a conditional inactivation of the *Apc* gene via a Cre-recombinase-driven excision of exon 14 under the control of intestine-specific human homeobox 2 (*CDX2*) promoter/enhancer (*CDX2P9.5-NLS Cre*). The *CDX2P9.5-NLS Cre*; *Apc^loxP/loxP^ (CPC;Apc^fl/fl^)* GEM model has been previously characterized [34]. The Cre-recombinase-mediated deletion of exon 14 of the mouse *Apc* gene in this GEM model creates a frameshift mutation at codon 580, resulting in the expression of a truncated APC protein of 605 amino acid residues in small intestinal and colon tissues [38].

The IHC analysis of KLK6 expression in the tumor tissue samples of *Apc^Min/+^* mice and KLK6 expressing *CPC;Apc^fl/fl^* mice (*CPC;Apc^fl/fl^;Klk6^+/+^*) confirmed a similar pattern of KLK6 protein localization in the luminal and epithelial areas of the tumors in both mouse models (Figure S2).

**Figure 3:**
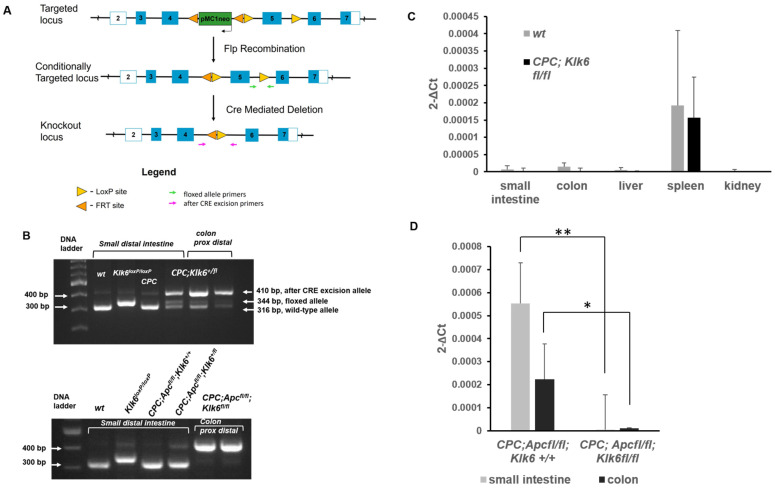
Establishment of *Klk6* gene conditional knockout mouse model and analysis of *Klk6* gene expression in the *CPC;Klk6^fl/fl^* and *CPC;Apc^fl/fl^;Klk6^fl/fl^* mice by qRT-PCR. (**A**) Schematic representation of the conditionally targeted allele of mouse *Klk6* gene and the truncated *Klk6* allele after deletion mediated by *Cre-loxP* recombination (knockout locus). (**B**) PCR genotyping analysis to confirm Cre-recombinase-mediated excision of targeted Klk6 sequence in the small intestinal tract and the colon of a compound *CPC;Apc^fl/fl^;Klk6^fl/fl^* mouse. PCR analysis results of wild-type mice, *Klk6^loxP/loxP^*, CPC, and *CPC;Klk6^+/fl^* mice confirmed Cre-recombinase-mediated excision of the one floxed allele in the *CPC;Klk6^+/fl^* mouse. Genotyping results of the wild-type, *Klk6^loxP/loxP^*, *CPC;Apc^fl/fl^;Klk6^+/+^*, and *CPC;Apc^fl/fl^;Klk6^fl/fl^* mice. A complete excision of the floxed *Klk6* region is seen in the analyzed portions of the intestines and colon of the homozygous mice *(CPC;Apc^fl/fl^;Klk6^fl/fl^*). (**C**) *Klk6* mRNA levels in the small intestine, colon, liver, spleen, and kidney of the 2-month-old *C57Bl6* mice (*wt*) and *Klk6^loxP/loxP^* mice after Cre recombinase-mediated excision of *Klk6* exon 5 (*CPC*; *Klk6^fl/fl^)* (n = 4 per group). (**D**) *Klk6* transcripts levels in the small intestines and the colons of the 6-month-old *CPC;Apc^fl/f^;Klk6^+/+^* (n = 7) and *CPC;Apc^fl/fl^;Klk6^fl/fl^* (n = 8) mice. Data are presented as the normalized average *Klk6* gene expression per mouse 2^−ΔCt^. * *p* = 0.027, ** *p* = 0.009 by non-parametric Kruskal–Wallis test.

A mouse with conditional *Klk6* alleles (*Klk6^loxP/loxP^*) was developed by inserting *loxP* sequences around the fifth exon of the mouse *Klk6* gene, corresponding to the third coding exon, since exons 1 and 2 are untranslated. The third coding exon encodes for the aspartic acid (D) residue in the KLK6 catalytic triad (histidine, aspartic acid, and serine) (Figure 3A). After Cre-recombinase mediated excision of the third coding exon in the mouse *Klk6* gene, only 72 amino acids are transcribed from the coding exons 1 and 2. Removal of the third coding exon shifts the three translation frames and introduces a stop codon in the first frame, leading to premature termination of the protein.

The *Klk6^loxP/loxP^* mice had a normal appearance on visual inspection, although a delay in fur growth was noted. Particularly, the 27 day-old *Klk6^loxP/loxP^* mice did not have fully grown fur, while their wild-type littermates completed fur growth by day 10 after birth (Figure S3). The *Klk6^loxP/loxP^* mice had fully grown fur by 8 weeks of age. The observed phenotype indicates that the loxP sequences introduced in the *Klk6* gene generated the hypomorphic *Klk6* alleles, leading to the transient partial loss of *Klk6* gene function in the hair follicles, where it is normally expressed [39]. No difference was observed in body weights between the *Klk*6 wild-type and *Klk6^loxP/loxP^* mice.

To achieve the intestinal tract-specific disruption of the *Klk6* gene in the *Apc* wild-type mouse, *Klk6^loxP/loxP^* mice were bred with *CPC* mice to create the *CPC;Klk6 ^fl/fl^* genotype. *Klk6^loxP/loxP^* mice were also crossed with *Apc^loxP/loxP^* mice, and the compound *Apc^loxP/loxP^; Klk6^loxP/loxP^* mice were crossed with *CPC* mice to create a mouse with biallelic inactivation of both *Apc* and *Klk6* genes, a *CPC;Apc^fl/fl^;Klk6^fl/fl^* mouse. The examples of the PCR analysis of a targeted *Klk6* sequence using *Klk6* region-specific primers, in the *Apc* and *Klk6* wild-type (wt), *Klk6^loxP/loxP^*, *CPC*, and *CPC;Klk6^+/fl^* mice, as well as in *CPC;Apc^fl/fl^* mice with the wild-type *Klk6* alleles (*CPC;Apc^fl/fl^;Klk6^+/+^*) and with biallelic inactivation of *Klk6* gene (*CPC;Apc^fl/fl^;Klk6^fl/fl^*), are shown in Figure 3B (upper and lower panels, respectively).

Analysis of *Klk6* RNA expression in *CPC;Klk6^fl/fl^*, *CPC;Apc^fl/fl^;Klk6^+/+^*, and *CPC;Apc^fl/fl^;Klk6^fl/fl^* mice was done by qRT-PCR. *Klk6* mRNA was rarely detected at low copy number levels or not detected in the small intestine, colon, liver, and kidney of the 2-month-old wild-type C57BL/6 and *CPC;Klk6^fl/fl^* mice. *Klk6* mRNA in the spleen was seen at a comparable level in both wild-type and *CPC;Klk6^fl/fl^* mice (Figure 3C). By contrast, *Klk6* mRNA transcript levels were detectable in the microscopically normal small intestine and colon tissue of 6-month-old *CPC;Apc^fl/fl^;Klk6^+/+^* mice. *Klk6* expression was significantly lower in the small intestine (*p* = 0.009) and colon (*p* = 0.027) of *CPC;Apc^fl/fl^;Klk6^fl/fl^* mice (Figure 3D). We also observed the intestinal region-specific difference in *Klk6* expression, that is, *CPC;Apc^fl/fl^;Klk6^+/+^* mice had a higher level of *Klk6* mRNA in the small intestine as compared to the colon, although this difference was not statistically significant due to the high variability in *Klk6* transcript levels in these mice.

### 3.4. Analysis of Histopathology and Tumorigenesis in CPC;Apc^fl/fl^;Klk6^+/+^ CPC;Apc^fl/fl^;Klk6^+/fl^ and CPC;Apc^fl/fl^;Klk6^fl/fl^ Mice

*CPC;Apc^fl/fl^;Klk6^+/+^* mice and their littermates with *Klk6* deleted were chosen for the study at 6 weeks of age after completion of genotyping. Mice were euthanized at the age of 6 months, or earlier if they became moribund due to severe anemia from intestinal hemorrhage or intestinal obstruction caused by the development of adenomas.

**Figure 4:**
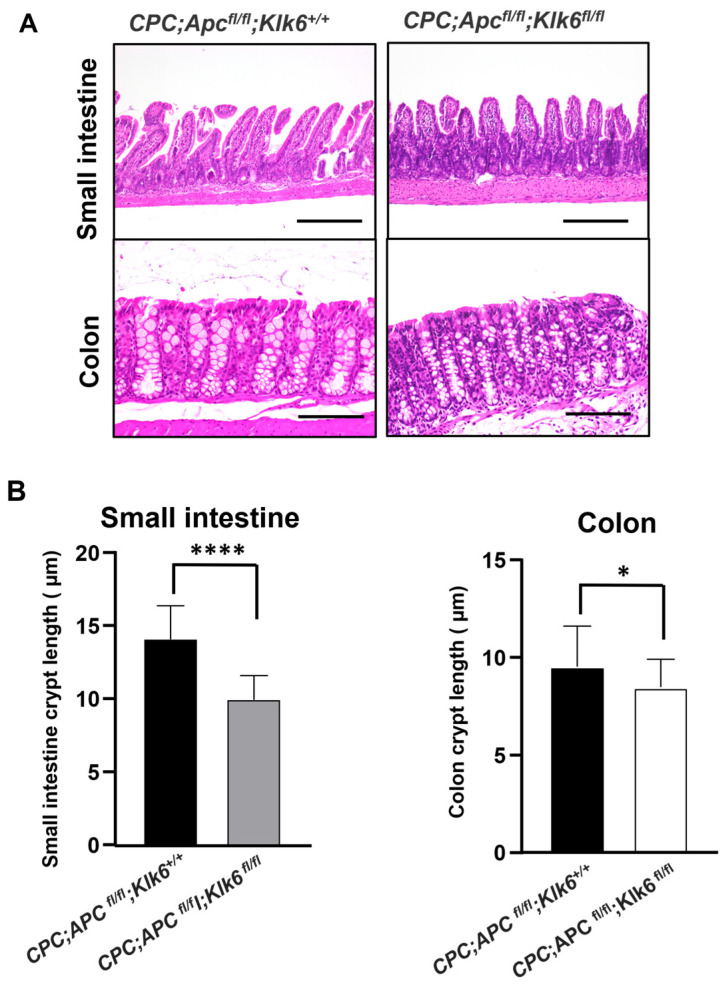
Histological analysis of the small intestines and colons of the 6-month-old *CPC;Apc^fl/fl^;Klk6^+/+^* animals with wild-type and disrupted *Klk6* genes. (**A**) Intestinal tract morphology of the *CPC;Apc^fl/fl^;Klk6^+/+^* and *CPC;Apc^fl/fl^;Klk6^fl/fl^* mice. The representative images of the regions in the distal small intestine and the distal colon of *CPC;Apc^fl/fl^;Klk6^+/+^* mice and *CPC;Apc^fl/fl^;Klk6^fl/fl^* mice are shown (Scale bar 200 μm). (**B**) Quantitation of crypt length (μm) in the small intestine and colon of the 6-month-old *CPC;Apc^fl/fl^;Klk6^+/+^* (n = 2) and *CPC;Apc^fl/fl^;Klk6^fl/fl^* (n = 4) mice. The average length of the small intestinal and colon crypts in *CPC;Apc^fl/fl^;Klk6^+/+^* mice (30 crypts per location) and *CPC;Apc^fl/fl^;Klk6^fl/fl^* mice (60 crypts per location) is shown. **** *p* < 0.0001, * *p* = 0.04 by non-parametric Mann–Whitney test.

The histological evaluation of the intestinal tract morphology of the 6-month-old *CPC;Apc^fl/fl^;Klk6^+/+^* and *CPC;Apc^fl/fl^;Klk6^fl/fl^* mice was performed using the H&E stained sections of the microscopically normal portions of the distal small intestine and the distal colon. The intestinal morphology and crypt appearance in *CPC;Apc^fl/fl^;Klk6^+/+^* mice with wild-type and inactivated *Klk6* genes are shown in Figure 4A. We performed the analysis of the small intestinal and colon crypt length in the randomly scored crypts of two *CPC;Apc^fl/fl^;Klk6^+/+^* mice and four *CPC;Apc^fl/fl^;Klk6^fl/fl^* mice (15 crypts per sample). Mice with homozygous disruption of the *Klk6* gene had significantly shorter small intestinal crypts (10.02 ± 1.66 μm) compared to *CPC;Apc^fl/fl^;Klk6^+/+^* mice, which expressed KLK6 (14.15 ± 2.32 μm) (Figure 4B, Small intestine, *p* < 0.0001). Similarly, *CPC;Apc^fl/fl^;Klk6^fl/fl^* mice had shorter colon crypts compared to *CPC;Apc^fl/fl^;Klk6^+/+^* mice (8.48 ± 1.43 μm versus 9.52 ± 2.08 μm, respectively) (Figure 4B, Colon, *p* = 0.04).

**Figure 5:**
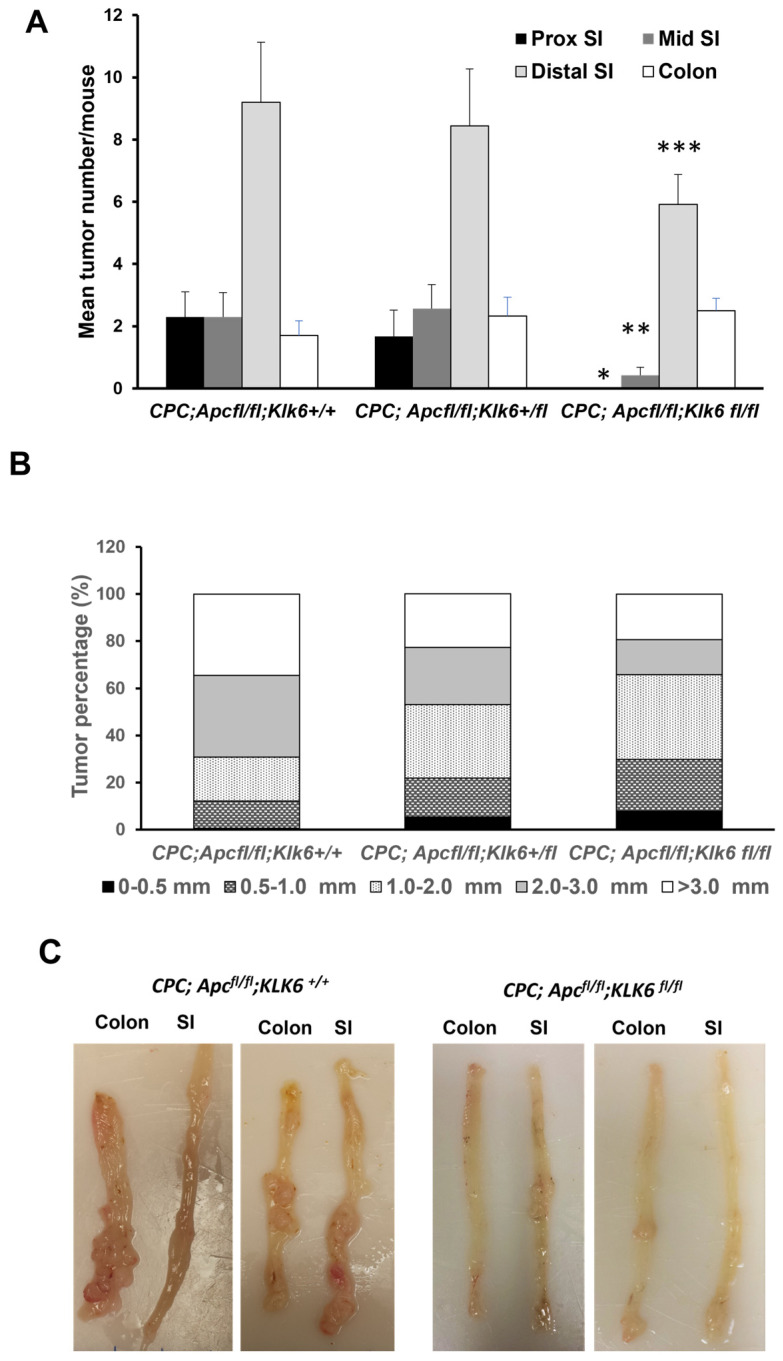
Effect of *Klk6* gene disruption in the small intestine and colon on *Apc*-mutant tumorigenesis. (**A**) Tumor counts in the small intestine and colon tissues of the *CPC;Apc^fl/fl^;Klk6^+/+^*, *CPC;Apc^fl/fl^;Klk6^+/fl^*, and *CPC;Apc^fl/fl^;Klk6^fl/fl^* mice. Tumor number was determined as described in the Materials and Methods section. Poisson regression analysis was used to calculate *p*-values between the genotypes (* *p* = 0.04 proximal portion, ** *p* = 0.03 middle portion, *** *p* = 0.02 distal portion of the small intestine, *CPC;Apc^fl/fl^;Klk6^+/+^* (n = 10) vs. *CPC;Apc^fl/fl^;Klk6^fl/fl^* (n = 12)). (**B**) Tumor size analysis in the *CPC;Apc^fl/fl^* mice with a different *Klk6* gene genotype. The size of adenomas was determined as described in the Materials and Methods section, and adenomas were separated into four groups according to their diameter in millimeters. Data are presented as the percentage of tumors in a certain size range from the total number of tumors in mice of the different genotypes. Data were analyzed using the chi-square test. (**C**) Representative images of the longitudinally dissected colons and distal small intestinal tissues representative of 6-month-old male and female *CPC;Apc^fl/fl^;Klk6^+/+^* and *CPC;Apc^fl/fl^;Klk6^fl/fl^* mice. *CPC;Apc^fl/fl^;Klk6^+/+^* mice had a visibly greater number of tumors throughout the small intestine and colon specimens.

We further examined how the intestine-specific *Klk6* gene inactivation affects the *Apc*-mutant tumorigenesis by measuring the number, the size, and the histological grade of the adenomas in the small intestines and colons of *CPC;Apc^fl/fl^;Klk6^+/+^* animals with the wild-type *Klk6* gene (*CPC;Apc^fl/fl^;Klk6^+/+^*) and in animals with heterozygous (*CPC;Apc^fl/fl^;Klk6^+/fl^*) or homozygous (*CPC;Apc^fl/fl^;Klk6^fl/fl^*) deletion of the gene. The average number of adenomas in the proximal, middle, and distal portions of the small intestine and the colon of animals of three genotypes are shown in Table 1. In the proximal small intestine, the *CPC;Apc^fl/fl^;Klk6^+/+^* and the *CPC;Apc^fl/fl^;Klk6^+/^^fl^* mice had a comparable number of adenomas (2.30 ± 2.67 and 1.67 ± 2.55, respectively), while remarkably no adenomas were observed in *CPC;Apc^fl/fl^;Klk6^fl/fl^* mice; Table 1 and Figure 5A (* *p* = 0.04, small intestine *CPC;Apc^fl/fl^;Klk6^+/+^* versus *CPC;Apc^fl/fl^;Klk6^fl/fl^*). In the middle and distal small intestine, *CPC;Apc^fl^^/fl^;Klk6^fl/fl^* mice had significantly fewer adenomas than *CPC;Apc^fl/fl^;Klk6^+/+^* mice (*p* = 0.03 and *p* = 0.02, respectively), while no significant difference was observed in the number of adenomas between the *CPC;Apc^fl/fl^;Klk6^+/+^* and *CPC;Apc^fl/fl^;Klk6^+/fl^* mice (*p* = 0.86 and *p* = 0.87, respectively). In the colon, no significant difference in the number of adenomas between genotypes was found (*p* = 0.39). Overall, the total number of adenomas per mouse was significantly higher in the *CPC;Apc^fl/fl^;Klk6^+/+^* mice (15.5 ± 4.22) and *CPC;Apc^fl/fl^;Klk6^+/fl^* mice (15.00 ± 5.47) than in the *CPC;Apc^fl/fl^;Klk6^fl/fl^* mice (8.83 ± 4.78) (*p* = 0.005). These data suggest that *Klk6* gene inactivation reduced tumor development in the new *CPC;Apc^fl/fl^;Klk6^fl/fl^* mouse model.

We evaluated the effect of KLK6 expression on the size of the tumors formed in *Apc*-mutant mice. The analysis revealed the difference between *CPC;Apc^fl/fl^;Klk6^+/+^* mice and mice heterozygous or homozygous for the *Klk6* gene in adenoma size distribution (Figure 5B). Specifically, the higher percentage of adenomas with sizes 2.0–3.0 mm and above 3.0 mm was seen in *CPC;Apc^fl/fl^;Klk6^+/+^* mice compared to the *CPC;Apc^fl/fl^;Klk6^+/fl^* and *CPC;Apc^fl/fl^;Klk6^fl/fl^* mice (*p* = 0.0018 and *p* = 0.0001, respectively). At the same time, the two-fold increase in the percentage of adenomas less than 2 mm was observed in the mice of *CPC;Apc^fl/fl^;Klk6^+/fl^* and *CPC;Apc^fl/fl^;Klk6^fl/fl^* genotypes (31.25% and 35.96%, respectively) compared to *CPC;Apc^fl/fl^;Klk6^+/+^* mice (18.68%). Figure 5C demonstrates the macroscopic view of the distal small intestine and the colon in *CPC;Apc^fl/fl^* mice with the wild-type and inactivated *Klk6* gene. This finding suggests that the inactivation of the *Klk6* gene in *Apc*-mutant animals leads to a delay in tumorigenesis progression, indicating that impaired KLK6 protease activity may slow down the development of tumors.

Histopathology analysis of cellular dysplasia in the adenomas of *CPC;Apc^fl/fl^;Klk6^+/+^*, *CPC;Apc^fl/fl^;Klk6^+/fl^*, and *CPC;Apc^fl/fl^;Klk6^fl/fl^* mice showed that the number of low-grade adenomas in the small intestine was similar in mice with different zygosity of the *Klk6* gene, ranging from 1.5 to 2 adenomas/mouse (Table 2 and Table 3, *p* = 0.65). In contrast, the number of high-grade adenomas in the small intestine of *CPC;Apc^fl/fl^;Klk6^fl/fl^* mice (0.58 ± 0.80) was significantly lower than in *CPC;Apc^fl/fl^;Klk6^+/+^* mice (1.7 ± 0.95) and *CPC;Apc^fl/fl^;Klk6^+/fl^* mice (1.71 ± 1.38) (*p* = 0.03 and *p* = 0.04, respectively). No statistically significant difference in the number of low-grade or high-grade adenomas in the colon was found between mice with differing zygosities of the *Klk6* gene (Table 2 and Table 3).

The pathology analysis revealed that *CPC;Apc^fl/fl^;Klk6^+/+^*, *CPC;Apc^fl/fl^;Klk6^+/fl^*, and *CPC;Apc^fl/fl^;Klk6^fl/fl^* mice had mild subacute multifocal colitis with mixed lymphocytic and neutrophilic infiltrates in the colon but not in the small intestine. The analysis of severity of inflammation using a lesion scoring system for assessment of the severity and extent of inflammation [36] revealed no statistically significant difference in inflammation scores between genotypes in the colon. The inflammation score was zero in the small intestines of all genotypes (Figure S4).

### 3.5. Analysis of Molecular Endpoints in CPC;Apc^fl/fl^;Klk6^+/+^, CPC;Apc^fl/fl^;Klk6^+/fl^, and CPC;Apc^fl/fl^;Klk6^fl/fl^ Mice

**Figure 6:**
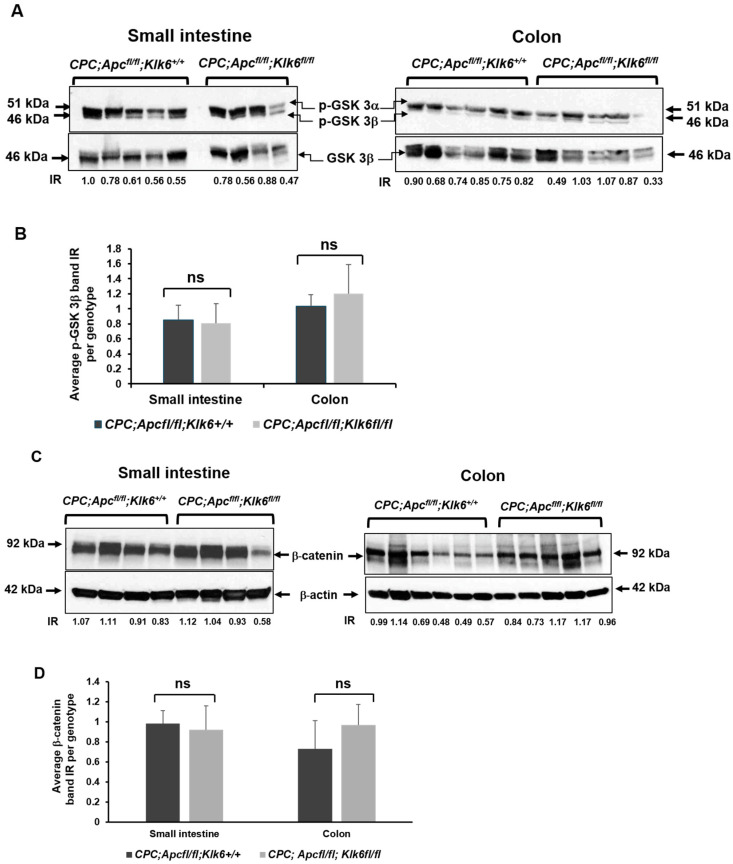

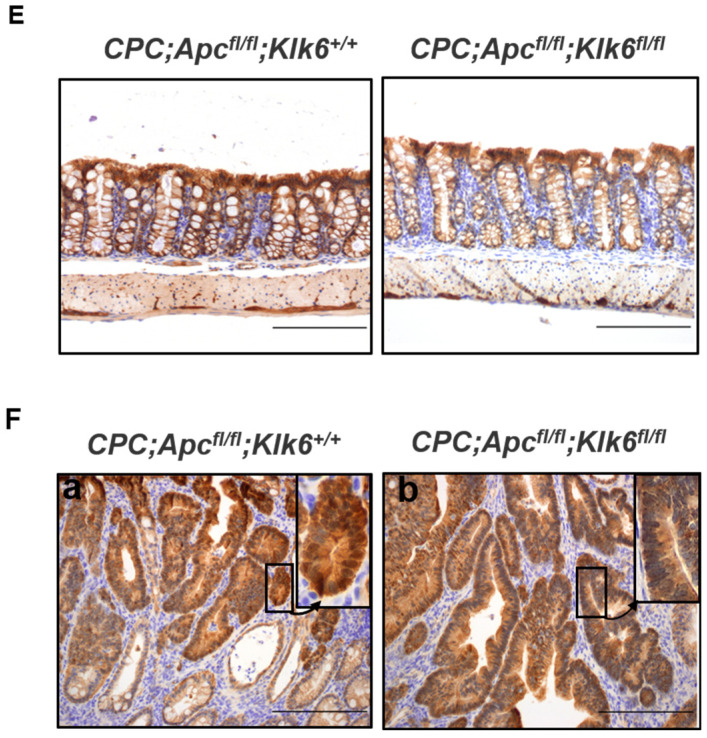
Analysis of Wnt signaling effectors in the small intestine and the colon of 6-month-old *CPC;Apc^fl/fl^;Klk6^+/+^*and *CPC;Apc^fl/fl^;Klk6^fl/fl^* mice. Each lane in subfigure (**A**) and subfigure (**C**) represents a different animal of the *CPC;Apc^fl/fl^;Klk6^+/+^* (n = 5, small intestine; n = 6, colon) and *CPC;Apc^fl/fl^;Klk6^fl/fl^* (n = 4, small intestine; n = 5, colon) genotypes. Data were analyzed using the ANOVA single-factor test (ns—*p* value not significant). The figure is representative of two independent experiments. (**A**) Western blot analysis of the total GSK3β protein and its phosphorylated form (p-GSK 3α/3β) in *CPC;Apc^fl/fl^;Klk6^+/+^* and *CPC;Apc^fl/fl^;Klk6^fl/fl^* mice. Representative cropped images of p-GSK 3α/3β and the total GSK3β derived from the original blots in Figure S5A. The intensity of the p-GSK 3α/3β and GSK3 3β bands in the small intestine and the colon of each animal was measured using ImageJ. The intensity ratios (IRs) of p-GSK 3α/3β to GSK 3β bands for each lane are included. (**B**) Graph represents the average normalized p-GSK 3α protein level in mice of *CPC;Apc^fl/fl^;Klk6^+/+^* and *CPC;Apc^fl/fl^;Klk6^fl/fl^* genotypes. (**C**) Evaluation of β-catenin protein level in the tissue lysates of *CPC;Apc^fl/fl^;Klk6^+/+^* and *CPC;Apc^fl/fl^;Klk6^fl/fl^* mice by Western blotting. β-actin was used as a loading control. Representative cropped images of β-catenin and β-actin derived from the original blots in Figure S5B. The intensity of β-catenin and β-actin bands in the small intestine and the colon of each animal was measured using ImageJ. The intensity ratios (IRs) of β-catenin and β-actin bands for each lane are included. (**D**) Graph represents the average normalized β-catenin protein levels in mice of *CPC;Apc^fl/fl^;Klk6^+/+^* and *CPC;Apc^fl/fl^;Klk6^fl/fl^* genotypes. (**E**) β-catenin immunostaining in the normal distal small intestine and normal distal colon tissue of *CPC;Apc^fl/fl^;Klk6^+/+^* and *CPC;Apc^fl/fl^;Klk6^fl/fl^* mice by IHC (Scale bar 200 μm). (**F**) Localization of β-catenin in small intestinal tumors of *CPC;Apc^fl/fl^;Klk6^+/+^*and *CPC;Apc^fl/fl^;Klk6^fl/fl^* mice by IHC (Scale bars 200 μm). Image (**a**) demonstrates strong nuclear and cytosolic accumulation of β-catenin in the tumor cells of the *CPC;Apc^fl/fl^;Klk6^+/+^* mouse; image (**b**) demonstrates the less intense β-catenin staining in the tumor cells of the *CPC;Apc^fl/fl^;Klk6^fl/fl^* mouse. The inserts show a higher magnification of selected areas of stained tissue. The representative images of β-catenin staining in three animals per genotype are shown.

The APC protein is known to activate glycogen synthase kinase 3β(GSK 3β), which promotes β-catenin degradation through its phosphorylation [3]. The lack of APC tumor suppressor function due to mutation causes a rise in the β-catenin protein level in cells and its translocation to the nucleus, where it serves as a transcriptional coactivator of cell proliferation genes such as the *C-MYC* oncogene [40]. We assessed the level of GSK 3β and its phosphorylated form (p-GSK 3α/β) and the level of β-catenin in the small intestine and colon of the *CPC;Apc^fl/fl^;Klk6^+/+^* and *CPC;Apc^fl/fl^;Klk6^fl/fl^* mice. The levels of p-GSK 3a/β and β-catenin proteins were not significantly altered upon *Klk6* gene disruption in the microscopically normal small intestinal and colon tissues (Figure 6A–D). However, variations in levels of these proteins were noted among mice. Further analysis of β-catenin expression in *CPC;Apc^fl/fl^;Klk6^+/+^* and *CPC;Apc^fl/fl^;Klk6^fl/fl^* mice by IHC staining demonstrated mostly membranous/cytoplasmic β-catenin localization in the normal small intestine and colon epithelium of mice from both genotypes (Figure 6E). In contrast, β-catenin localization in the tumors of *CPC;Apc^fl/fl^;Klk6^+/+^* mice seems to be mostly in the nuclei (Figure 6(Fa)), while lesser β-catenin staining is seen in the nuclei of the tumors of *CPC;Apc^fl/fl^;Klk6^fl/fl^* mice (Figure 6(Fb)). Overall, the β-catenin staining in tumor samples of *CPC;Apc^fl/fl^;Klk6^+/+^* mice had the higher intensity score (average score of three) compared to the intensity of β-catenin staining in the tumors of *CPC;Apc^fl/fl^;Klk6^fl/fl^* mice (average score of two).

The c-MYC oncogene is an established downstream target gene of the Wnt-signaling pathway, and c-MYC in turn can induce a transcription of cell-growth-promoting gene ornithine decarboxylase (*ODC1*) [40,41]. We assessed the expression of the c-MYC protein in the normal small intestine and colon tissues of *CPC;Apc^fl/fl^;Klk6^+/+^* and *CPC;Apc^fl/fl^;Klk6^fl/fl^* mice by Western blotting. No significant differences were noted in the levels of c-MYC protein (Figure S6A, derived from original blots in Figure S6B). The *Odc1* mRNA level was measured in the mouse intestinal tract by qPCR, and no difference in *Odc1* expression was found between animals with the wild-type and disrupted *Klk6* gene (Figure S6C). To determine the effect of *Klk6* inactivation on cellular proliferation, we performed IHC staining for a cellular marker of cell proliferation, the Ki-67 protein, in the samples of the microscopically normal distal small intestine and distal colon in the 6-month-old mice of *CPC;Apc^fl/fl^;Klk6^+/+^* and *CPC;Apc^fl/fl^;Klk6^fl/fl^* genotypes. The Ki-67 IHC staining patterns were similar in mice of both genotypes (Figure S7A). The quantitative analysis of the intensity of staining showed no significant difference in Ki-67 expression between *CPC;Apc^fl/fl^;Klk6^+/+^* and *CPC;Apc^fl/fl^;Klk6^fl/fl^* mice (Figure S7B).

**Figure 7:**
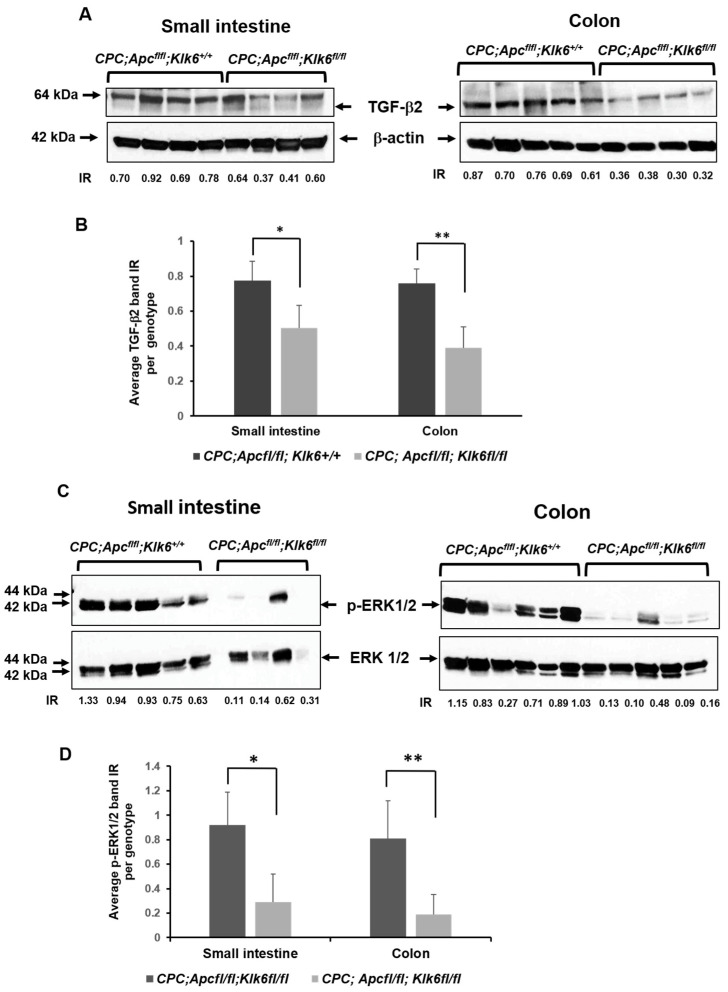

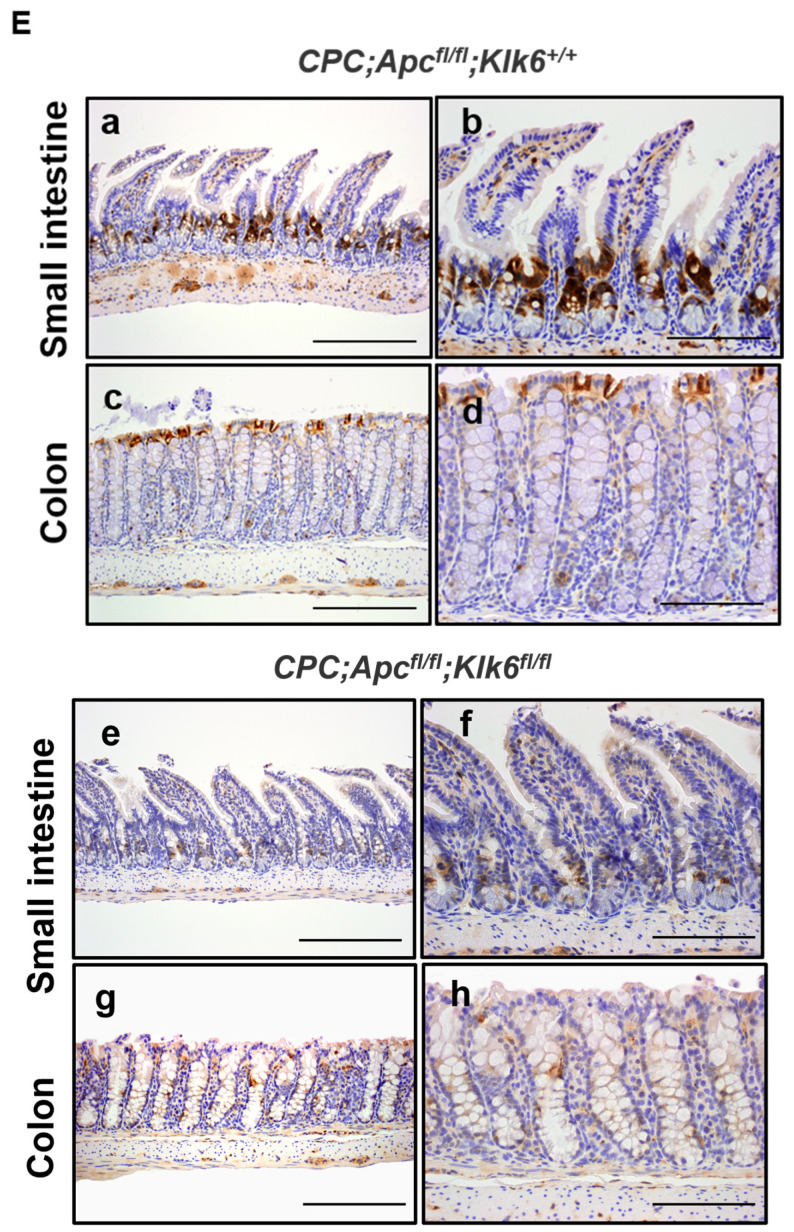
Analysis of TGF-β and the downstream signaling molecules in the small intestine and the colon of *CPC;Apc^fl/fl^;Klk6^+/+^* and *CPC;Apc^fl/fl^;Klk6^fl/fl^* mice. Each lane represents a different animal of the *CPC;Apc^fl/fl^;Klk6^+/+^* and *CPC;Apc^fl/fl^;Klk6^fl/fl^* genotypes. Data were analyzed by the ANOVA single factor test. The figure is representative of two independent experiments. (**A**) Western blot analysis of TGF-β2, protein level in *CPC;Apc^fl/fl^;Klk6^+/+^* mice (small intestine, n = 4; colon, n = 5) and *CPC;Apc^fl/fl^;Klk6^fl/fl^*, mice (small intestine n = 4; colon n = 4). β-actin was used as a loading control. Representative cropped images of TGF-β2 and β-actin derived from the original blots in Figure S9A. The intensities of TGF-β2 and β-actin bands in the small intestine and the colon of each animal were measured using ImageJ. The intensity ratios (TGF-β2/β-actin) for each band are included. (**B**) Graph represents the average normalized expression of TGF-β2 protein in the small intestine and the colon of *CPC;Apc^fl/fl^;Klk6^+/+^* and *CPC;Apc^fl/fl^;Klk6^fl/fl^* mice. * *p* = 0.02, ** *p* = 0.01. (**C**) Western blot analysis of phosphorylated form (p-ERK1/2) and the total ERK1/2 (ERK1/2) protein in the small intestine and the colon of *CPC;Apc^fl/fl^;Klk6^+/+^*and *CPC;Apc^fl/fl^;Klk6^fl/fl^* mice. Representative cropped images of p-ERK1/2 and ERK1/2 derived from the original blots in Figure S9B. The intensity of the p-ERK1/2 and ERK1/2 bands in the small intestine and the colon of each animal was measured using ImageJ. The intensity ratios (IRs) (p-ERK1/2/ERK1/2) for each band are included (*CPC;Apc^fl/fl^;Klk6^+/+^* mice: small intestine, n = 5, colon n = 6; *CPC;Apc^fl/fl^;Klk6^fl/fl^*, mice: small intestine n = 4, colon n = 5). (**D**) Graph represents the average normalized expression level of p-ERK1/2 protein in *CPC;Apc^fl/fl^;Klk6^+/+^* and *CPC;Apc^fl/fl^;Klk6^fl/fl^* mice. * *p* = 0.01, ** *p* < 0.01. (**E**) Distribution of p-ERK1/2 protein in the distal small intestine and distal colon of *CPC;Apc^fl/fl^;Klk6^+/+^* and *CPC;Apc^fl/fl^;Klk6^fl/fl^* mice evaluated by IHC. Images (**a**,**c**,**e**,**g**)—scale bar = 200 μm; images (**b**,**d**,**f**,**h**)—scale bar = 100 μm. The representative images of staining in three animals per genotype are shown.

As has been previously reported, elevated KLK6 expression can accelerate the EMT in colon cancer cells and can activate the signal transduction pathways controlling cell invasion and metastasis via the Transforming Growth Factor β2 (TGF-β2) signaling pathway [28,29]. We assessed the levels of TGF-β2 protein and its downstream effectors, SAMD2/3, as well as the epithelial cell marker E-cadherin proteins in the small intestines and colons of *CPC;Apc^fl/fl^;Klk6^+/+^* and *CPC;Apc^fl/fl^;Klk6^fl/fl^* mice. The *CPC;Apc^fl/fl^;Klk6^fl/fl^* mice had significantly lower levels of TGF-β2 in the small intestine and colon compared to *CPC;Apc^fl/fl^;Klk6^+/+^* mice (Figure 7A,B, *p* = 0.02 and *p* = 0.01, respectively). At the same time, the levels of phosphorylated SMAD2 (p-SMAD2) protein and E-cadherin protein were similar in mice of both genotypes (Figure S8A and S8B, respectively). This indicates that KLK6 is likely not directly involved in TGF-β2-SMAD signaling to promote intestinal tumorigenesis in the *CPC;Apc^fl/fl^* mouse model.

The TGF-β signaling pathways and the mutant *Apc* gene both can activate extracellular signal-regulated kinase 1/2 (ERK1/2) mitogen-activated protein kinase (MAPK) during intestinal tumorigenesis [42,43]. We performed Western blot analysis of phosphorylated (Thr202/Tyr204) and total ERK1/2 in *CPC;Apcf^l/fl^* mice with the wild-type and disrupted *Klk6* gene (Figure 7C). The *CPC;Apc^fl/fl^;Klk6^fl/fl^* mice had significantly lower levels of the phosphorylated ERK1/2 (p-ERK1/2) in the normal small intestinal and colon tissues as compared to KLK6-expressing *CPC;Apc^fl/fl^;Klk6^+/+^* mice (Figure 7D, *p* = 0.01 and *p* ≤ 0.001, respectively).

As shown in Figure 7E, the IHC staining for p-ERK1/2 revealed the differences in the distribution of p-ERK protein throughout the normal intestine and colon epithelium of the *Apc*-mutant mice. The p-ERK1/2 staining was localized predominantly in the small intestinal crypt and the crypt/villi junction areas of the small intestine in the *CPC;Apc^fl/fl^;Klk6^+/+^* mice (Figure 7(Ea,Eb)). In the colon, the p-ERK1/2-positive cells were localized within the upper area of the normal mucosa (Figure 7(Ec,Ed)). Interestingly, the *CPC;Apc^fl/fl^;Klk6^fl/fl^* mice had significantly less staining for p-ERK1/2 in the small intestinal crypts than the *CPC;Apc^fl/fl^;Klk6^+/+^* mice (Figure 7(Ee,Ef)). Similarly lower levels of staining for p-ERK1/2 were observed in the colon (Figure 7(Eg,Eh)), suggesting that the inactivation of the *Klk6* gene may be associated with reduction of ERK1/2 pathway activation during CRC development.

## 4. Discussion

Kallikrein-related peptidase enzymes are involved in proteolytic cascades through their function in the degradation of extracellular matrix proteins and promotion of angiogenesis and, therefore, are implicated in several pathophysiological processes, including cancer [44]. We have previously shown the role of KLK6 in colon cancer cell invasion [28]. Using in vitro and in vivo colon cancer models, we also showed that KLK6 expression is upregulated by the mutant *K-RAS* oncogene [27,45,46]. However, the role of KLK6 in intestinal tumor development in vivo is still unknown.

*Klk6* is the mouse ortholog of human KLK6 that belongs to a large family of kallikrein-related peptidases, representing secreted serine proteinases with diverse expression patterns and functions in cell physiology, including cancer [44]. A comparison of the human and murine loci shows that the location of most genes in the kallikrein family is conserved, except for the genes encoding *KLK2* and *KLK3* (*PSA*), where the mouse carries pseudogenes [47]. The mouse analog of *KLK6* allows for the direct study of the role of KLK6 in various biological processes. Mouse models with the knockout of the *Klk6* gene have become a valuable tool for understanding KLK6 function in several human diseases (e.g., spinal cord injury, Parkinson’s disease, skin regeneration, Netherton syndrome) [48,49,50,51,52].

In this study, we found that *Klk6* gene expression was significantly elevated (up to four-fold higher compared to the microscopically normal tissue) in the adenomas formed in the intestinal tract of the *Apc^Min/+^* mouse model of the human familial adenomatous polyposis disease.

To understand the contribution of KLK6 to the colorectal tumorigenesis driven by the mutant *Apc* gene, we generated the *CPC;Apc^fl/fl^;Klk6^fl/fl^* mouse model with intestine- and colon-specific homozygous inactivation of the functional APC and KLK6 proteins through *CDX2P9.5-NLS Cre (CPC)*-driven Cre recombinase expression. We found that *Klk6* inactivation did not alter the overall morphology of the small intestine and colon in mice with wild-type APC. However, the *Apc*-mutant mice with the inactivated *Klk6* gene function had significantly shorter crypt lengths in the distal small intestine and colon (*p* < 0.0001 and *p* = 0.04, respectively).

The disruption of KLK6 enzyme function in *CPC;Apc^fl/fl^;Klk6^fl/fl^* mice resulted in the overall two-fold suppression of adenoma formation compared to the *CPC;Apc^fl/fl^;Klk6^+/+^* mice (*p* = 0.01). Interestingly, the *Klk6* disruption significantly decreased the percentage of adenomas larger than 2 mm (*p* ≤ 0.0018). In the meantime, the percentage of adenomas smaller than 2 mm increased by two-fold. Furthermore, the number of high-grade adenomas formed in the small intestine was significantly lower in the *CPC;Apc^fl/fl^;Klk6^fl/fl^* mice as compared to the *Klk6*-expressing *CPC;Apc^fl/fl^;Klk6^+/+^* mice (*p* = 0.03). These findings indicate that KLK6 expression promotes the formation of larger and more dysplastic tumors in the *APC*-mutant intestinal epithelium. This observation has a clinical significance, since a link has been established between the high-grade dysplasia in patients’ benign adenomas and the development of advanced colorectal neoplasia [53,54]. Thus, KLK6 overexpression in the colorectal tissue could be contributing to the formation of high-risk lesions in the adenoma-carcinoma sequence and can indicate early-stage carcinoma development.

The suppression of adenoma formation was evident in the small intestinal tract of the *CPC;Apc^fl/fl^;Klk6^fl/fl^* mice, but it did not reach statistical significance in the colon. This observation will require further investigation to understand the molecular mechanisms that may influence the tumorigenesis in this mouse model, including the higher *Klk6* transcript level in the small intestine versus in the colon in the *CPC;Apc^fl/fl^;Klk6^+/+^* mice and the contribution of *Apc* gene modifiers [32,55].

Regardless of the status of *Klk6* expression, the *Apc*-mutant mice had mild inflammation in the colon, while the inflammation was absent in the small intestines. The expression of the p65 subunit of the NF-κB transcription factor was not detectable in the small intestine of animals of both genotypes, which is consistent with the lack of small intestine inflammation by histopathology. The variable genotype-independent expression of NF-κB p65 protein was noted in the colon tissues of *CPC;Apc^fl/fl^;Klk6^+/+^* and *CPC;Apc^fl/fl^;Klk6^fl/fl^* mice. This finding is in agreement with the previously reported inhibitory effect of the mutant *Apc* on the NF-kB activity in the mutant APC-expressing human colon cell lines [56].

Wnt/β-catenin signaling is essential for intestinal homeostasis. In many colorectal cancers, β-catenin is not degraded properly because of mutation of the tumor suppressor *APC*, and it then facilitates transcription of cell proliferation genes or interacts with cell structural proteins and contributes to cell adhesion and migration (reviewed in [57]). Even though the level of the total β-catenin protein in tissue lysates was not significantly altered in the microscopically normal intestine and colon tissue samples upon *Klk6* gene disruption, the β-catenin IHC staining was less intense in the nuclei of the tumors of *CPC;Apc^fl/fl^;Klk6^fl/fl^* mice compared to the intensity of β-catenin staining in the tumors of *CPC;Apc^fl/fl^;Klk6^+/+^* mice.

The *c-MYC* oncogene is known to be elevated in the *Apc^Min/+^* mouse model and humans with FAP [58]. Conditional disruption of the *Klk6* gene in *CPC;Apc^fl/fl^;Klk6^fl/fl^* mice did not alter the c-MYC protein level in these mice. In agreement with these data, the cell proliferation marker Ki-67 also was not different in the intestinal tract of *CPC;Apc^fl/fl^;Klk6^fl/fl^* mice when compared to *CPC;Apc^fl/fl^;Klk6^+/+^* mice. This suggests that KLK6 does not contribute to cell proliferation in the *Apc*-mutant intestinal tract but may influence tumorigenesis via different molecular pathways that influence the tumor microenvironment.

We measured the levels of TGF-β2 and downstream effectors p-SMAD2 and SMAD2/3 proteins in the intestinal tract of the animal model with *Klk6* gene disruption. We found that TGF-β2 protein level was significantly lower in the small intestine and colon of *CPC;Apc^fl/fl^;Klk6^fl/fl^* mice as compared to *CPC;Apc^fl/fl^;Klk6^+/+^* mice. Surprisingly, the phosphorylation of the TGF-β2 downstream kinase SMAD2 was not significantly altered between the mice of *Klk6* wild-type and knockout genotypes. Nonetheless, Erk1/2 phosphorylation was significantly lower in *CPC;Apc^fl/fl^;Klk6^fl/fl^* mice compared to *CPC;Apc^fl/fl^;Klk6^+/+^* mice.

KLK6 and TGF-β are both involved in complex cellular processes, including cancer progression. It is known that different cancer signaling pathways, such as TGF-β, mutant *APC*, and an enzymatically active KLK6, lead to the activation of the ERK1/2 MAP kinase during intestinal tumorigenesis [30,42,43,59]. Particularly, KLK6 protease can activate PAR-1 and PAR-2, which can trigger MAPK phosphorylation [19,30,31]. The G protein-coupled receptors PAR1 and PAR2 are crucial factors in cancer development through protease signaling, and they have been linked to colon cancer cell proliferation and stimulation of cell migration and invasion [30,59,60,61]. The *Klk6* inactivation in our mouse model did not suppress cell proliferation while reducing MAPK phosphorylation specifically in the small intestinal crypts. The cessation of proliferation associated with the high MAPK activity and EMT has been reported specifically at the leading tumor edge in the *APC*-mutant CRC tumors [62]. This suggests that the KLK6 enzyme may contribute to cell migration/invasion through TGF-β-mediated MAPK and/or PARs activation directly or indirectly [63]. Further studies will help to determine functional interactions between TGF-β and KLK6 signaling, which can synergistically enhance tumorigenesis.

Overall the histopathology, tumorigenesis, and molecular analyses of the *CPC;Apc^fl/fl^;Klk6^fl/fl^* mice showed that *Klk6* gene disruption delayed the progression of tumorigenesis driven by the mutant *Apc*.

A previous analysis of the kallikrein-related peptidase family in CRC tumor samples from the TCGA database revealed that KLK6 overexpression in human tumors is associated with the overexpression of other members of the kallikreins protease family, i.e., KLK7, KLK8, and KLK10 [18]. Interestingly, these kallikreins have been reported to contribute to colorectal tumorigenesis by increasing cell migration and invasion via activation of PAR-1 and promoting the EMT [64,65]. Therefore, other active proteases could compensate for KLK6 protein function when the *Klk6* gene is disrupted in *CPC;Apc^fl/fl^;Klk6^fl/fl^* mice.

The current lack of approaches to specifically detect active KLK6 enzymes has constrained our studies to measuring the total *Klk6* mRNA level rather than assessing protein level or functional KLK6 activity. Developing probes to detect specific KLK6 activity would facilitate further investigation into the specific roles of KLK6 functionality in cancer progression and help to validate KLK6 as a potential drug target. Nevertheless, our ability to detect KLK6 protein in the adenomatous areas of the mouse colon using a highly accurate OCT/LEF imaging [37] with fluorophore-labeled KLK6 antibody demonstrated the potential future application of KLK6 as a tumor marker. The combination of OCT and LIF can provide detailed structural and biochemical information, making this method highly effective for intraoperative and post-surgical assessment of tumor margins [66]. The fact that KLK6 is associated with a more aggressive tumor phenotype and is detectable in colon adenomas offers a potentially valuable clinical tool in differentiating the more aggressive CRC tumors.

## 5. Conclusions

The overexpression of KLK6 has been reported in the adenomas of CRC patients [20]. Our study demonstrates that KLK6 inactivation in the mouse intestinal epithelium suppressed the number, size, and histological grade of intestinal adenomas through suppression of TGF-β and MAPK activity. Although the exact mechanism of upregulation of KLK6 and other proteases in the presence of *APC* mutations is not completely understood, our study contributes to the recognition of KLK6’s role in tumor development and demonstrates a possibility for using KLK6 for CRC detection and therapy.

## Figures and Tables

**Table 1:** Summary of total number of adenomas at each location for each genotype.

Location	Genotype	Animals/ Genotype	Mean ± SD	Range	% (>0) ^a^	*p*-Value ^b^	
Small Intestine (SI) Prox SI							
	*CPC;Apc^fl/fl^;Klk6^+/+^*	10	2.30 ± 2.67	0–6	50.00		
	*CPC;Apc^fl/fl^;Klk6^+/fl^*	9	1.67 ± 2.55	0–6	33.33	0.85	
	*CPC;Apc^fl/fl^;Klk6^fl/fl^*	12	0.00 ± 0.00	0–0	0.00	0.04	
Midd SI							
	*CPC;Apc^fl/fl^;Klk6^+/+^*	10	2.30 ± 2.58	0–6	50.00		
	*CPC;Apc^fl/fl^;Klk6^+/fl^*	9	2.55 ± 2.35	0–6	77.78	0.86	
	*CPC;Apc^fl/fl^;Klk6^fl/fl^*	12	0.41 ± 0.90	0–3	25.00	0.03	
Distal SI							
	*CPC;Apc^fl/fl^;Klk6^+/+^*	10	9.20 ± 6.41	3–22	100.00		
	*CPC;Apc^fl/fl^;Klk6^+/fl^*	9	8.44 ± 5.50	3–18	100.00	0.87	
	*CPC;Apc^fl/fl^;Klk6^fl/fl^*	12	5.92 ± 3.34	1–12	100.00	0.02	
Colon (CO)							
	*CPC;Apc^fl/fl^;Klk6^+/+^*	10	1.70 ± 1.56	0–5	70.00		
	*CPC;Apc^fl/fl^;Klk6^+/fl^*	9	2.33 ± 1.80	0–5	77.78	0.80	
	*CPC;Apc^fl/fl^;Klk6^fl/fl^*	12	2.50 ± 1.38	0–5	91.67	0.45	
Total (SI + CO)							
	*CPC;Apc^fl/fl^;Klk6^+/+^*	10	15.50 ± 4.22	11–24	100.00		
	*CPC;Apc^fl/fl^;Klk6^+/fl^*	9	15.00 ± 5.47	9–24	100.00	0.89	
	*CPC;Apc^fl/fl^;Klk6^fl/fl^*	12	8.83 ± 4.78	1–18	100.00	0.01	

^a^ Percent of mice with tumors; ^b^ *CPC;Apc^fl/fl^;Klk6^+/+^* vs. *CPC;Apc^fl/fl^;Klk6^+/fl^* or *CPC;Apc^fl/fl^;Klk6^fl/fl^*. Red color indicates a statistically significant *p*-value.

**Table 2:** Summary of total adenoma count by histopathology for each genotype.

Location	Genotype	Dysplasia	Mean (+/−SD)	Range	% (>0) ^a^
Small	*CPC;Apc^fl/fl^;Klk6^+/+^*	Low	2.00 (+/−1.56)	0–5	80.00
intestine		High	1.70 (+/−0.95)	1–4	100.00
	*CPC;Apc^fl/fl^;Klk6^+/fl^*	Low	1.85 (+/−1.57)	0–4	71.43
		High	1.71 (+/−1.38)	0–4	85.71
	*CPC;Apc^fl/fl^;Klk6^fl/fl^*	Low	1.50 (+/−0.80)	1–3	100.00
		High	0.58 (+/−0.80)	0–3	33.33
Colon	*CPC;Apc^fl/fl^;Klk6^+/+^*	Low	0.30 (+/−0.67)	0–2	20.00
		High	1.00 (+/−1.05)	0–3	60.00
	*CPC;Apc^fl/fl^;Klk6+^/fl^*	Low	0.14 (+/−0.38)	0–1	14.29
		High	1.28 (+/−1.25)	0–3	71.43
	*CPC;Apc^fl/fl^;Klk6^fl/fl^*	Low	0.25 (+/−0.45)	0–1	25.00
		High	0.75 (+/−1.05)	0–3	41.67

^a^ Percent of mice with tumors.

**Table 3:** Statistical significance (*p*-value) of difference in total number of adenomas by histopathology between genotypes.

		*CPC;Apc^fl/fl^;Klk6^+/+^* Versus *CPC;Apc^fl/fl^;Klk6^+/fl^*	*CPC;Apc^fl/fl^;Klk6^+/+^* Versus *CPC;Apc^fl/fl^;Klk6^fl/fl^*	*CPC;Apc^fl/fl^;Klk6^+/fl^* Versus *CPC;Apc^fl/fl^;Klk6^fl/fl^*
Small	Low Grade	0.83	0.37	0.56
intestine	High Grade	0.98	0.03	0.04
Colon	Low Grade	0.50	0.82	0.72
	High Grade	0.58	0.53	0.26

Red color indicates a statistically significant *p*-value.

## Data Availability

The data that support the fundings of this study are available from the corresponding author (N.A.I.) upon reasonable request. The *Klk6 cKO* mouse model will be available in Mouse repository NCI-Frederick upon approval and completion of the strain submission request.

## Supplementary Materials

The following supporting information can be downloaded at: https://www.mdpi.com/article/10.3390/cancers16223842/s1, Supplementary Materials and Methods, Figure S1. Validation of the specificity of a human-specific KLK6 antibody in the mouse tissue; Figure S2. Representative images of KLK6 IHC staining with human-specific KLK6 antibody; Figure S3. Example of a delay in fur growth in the 27-day-old C57BL6 *Klk6^loxP/loxP^* mice; Figure S4. Average inflammation scores in the colon of animals; Figure S5. A. The uncropped Western blot images of p-GSK3α/3β and GSK3β proteins presented in Figure 6A. Molecular weight (MW) markers are shown on the left side of the images. B. The uncropped Western blot images of β-catenin and β-actin proteins presented in Figure 6C. Molecular weight (MW) markers are shown on the β-catenin image; Figure S6. Evaluation of c-MYC and ODC expression in *CPC;Apc^fl/fl^* mice with different status of the *Klk6* gene; Figure S7. Ki-67 protein expression in the small intestine and colon of *CPC;Apc^fl/fl^* and *CPC;Apc^fl/fl^;Klk6^fl/fl^* mice was analyzed by IHC; Figure S8. SMAD signaling is not altered in *CPC;Apc^fl/fl^* mice upon disruption of *Klk6* gene; Figure S9. A. The uncropped Western blot images of TGF-β2 and β-actin proteins presented in Figure 7A. Molecular weight (MW) markers are shown on the TGF-β2 image. B. The uncropped Western blot images of p-ERK1/2 and ERK1/2 proteins presented in Figure 7C. Molecular weight (MW) markers are shown on the left of the images. Table S1. List of antibodies used in Western blotting.

## Abbreviations

CRC: colorectal cancer; *APC*: *Adenomatous polyposis coli*; KLK6: Kallikrein 6; GEM: genetically engineered mouse; *cKO*: conditional knockout; OCT/LIF: optical coherence tomography/laser-induced fluorescence; TGF-β2: transforming growth factor β2 isoform; GSK3β: glycogen synthase kinase 3β; ODC: ornithine decarboxylase; MAPK: p44/42 mitogen-activated protein kinase; IHC: immunohistochemistry; EMT: epithelial–mesenchymal transition.
