# Supplementary material for: Kallikrein-Related Peptidase 6 Contributes to Murine Intestinal Tumorigenesis Driven by a Mutant Adenomatous polyposis coli Gene
Source: Cancers (Basel). 2024 Nov 15;16(22):3842. doi: 10.3390/cancers16223842 (PMC11592602; doi:10.3390/cancers16223842)
Supplement: Supplementary file 1 [file cancers-16-03842-s001.zip › cancers-3313527-supplementary.pdf]

Kallikrein-related peptidase 6 contributes to murine intestinal tumorigenesis driven by a mutant *Adenomatous Polyposis Coli* gene

SUPPLEMENTARY MATERIALS

Supplementary Materials and Methods

Section: Generation of *Klk6* conditional knockout mouse model

A gene targeting vector with a floxed *Klk6* allele was designed with *loxP* sites flanking the fifth exon (3rd coding exon) of the mouse *Klk6* gene (which corresponds to exon 3 in a human *KLK6* gene). The 2.6 kb left arm of homology was generated by PCR amplification of genomic C57BL/6J DNA. The primers incorporated unique 5' Asc I and 3' Sac II sites to facilitate directional cloning in front of flippase (FLP recombinase) recognition target (FRT) sites that flanked the MC1 neo and *loxP* containing pcKOB vector (kind gift of Yinhuai Chen, University of Cincinnati). The middle arm of homology (1.62 kb) was generated in a similar fashion as the left arm. The primers incorporated a unique Cla I site on the 5' end and unique Sal I site along the loxP sequence on the 3' end to assure directional incorporation into the targeting vector. The right arm or long arm of homology (3.15 kb) used 5' primers with the Sal I site and 3' primers with a unique Pac I site for ligation into the conditional targeting vector. The total homology in this vector was 5.75 kb. Bruce 4 mouse ES cells (Millipore) were electroporated in the presence of 4.1 ug of Pac I linearized vector. G418 and gancyclovir drug resistant clones were picked up and screened for the presence of the targeted vector. Positive clones were identified by outside:inside PCR screening, where outside represents primers outside of arms of homology, and inside represents PCR primers in the neo cassette. Additionally, the *loxP* sites were sequenced to ensure their presence and intactness. Targeted ES cell clones were microinjected into B6D2xBL6 blastocysts and implanted into pseudo

pregnant ND4 Swiss Webster females (Envigo, Indianapolis, IN). Male 100% chimeras were bred to C57BL/6 females.

Section: Mouse *Klk6* gene conditional knockout primers

Germline transmission was detected by tail PCR screening using the following primers: the forward primer: **GGGCAGACCTGAGCATCC**, reverse primer **GGTTATCTGGCTTGTGACACC,** and neo reverse primer **CCCAAGTTCGGGTGAAGG** (the targeted vector yields 444 bp PCR product and the intact allele yields 364 bp product).

F2 generation mice were crossed with the Flp recombinase expressing mouse strain, *B6.Cg-Tg(ACTFLPe)920Dym/J* (JAX Stock Number 005703) to remove the *neo* sequence. Screening for the neo-excised targeted allele was done using the following primers: forward **GGGCAGACCTGAGCATCC** and reverse **GGTTATCTGGCTTGTGACACC**, producing a 444 bp PCR product for the wild-type allele and 559 bp one for the targeted allele. (The fragment from the targeted allele is bigger because it includes the one loxP and a few base pairs from the vector including the restriction sites). The sequencing data for *Klk6* wild type, targeted allele and after Cre-recombinase mediated excision are presented below.

Before Cre excision, the targeted allele PCR product is longer than the wild-type one due to the presence of the loxP site: 344bp for the floxed allele vs 316bp for the wild-type allele.

Mouse KLK6 gene wild-type sequence and genotyping primers

```
CATCATGATGGTGCATCTGAAAAATCCAGTCAAATTCTCTAAAAAGATCCAGCC
AGAATCCCAACTGCAGATCCTGGGCTGGGGCAAGAT
GGAAAATG
gtcagtgaggggagatggttgggagtgatcaaagggaccaaagggagagggtctagtgat 316bp
ggaggtgggatggtaaatagtgaaggtgggaaagaagatggtgggccacaaggagatgct
gatggagaggatcagtaggtcactctgtttatggataaaagacagatggagagaggagaa
ggttgaggagttggaccaaatgtggataggctacaaaatgaagggtcaatgagaagtgag
aactcatgggggaggttatgattagtgaggaagtcagaccgagaatgatggggaaagggg
```

Mouse *Klk6* targeted allele sequence and genotyping primers

```
CTTGAAGAATGACTGCTCTGAGGAGAATCCCAACTGCCAGATCCTGGGCTGGGGCAAGAT
GGAAAATGgtcagtgaggggagatggttgggagtgatcaaagggaccaaagggagaggg
tctagtgatggaggtgggatggtaaatagtgaaggtgggaaagaagatggtgggccacaa
ggagatgctgatggagaggatcagtaggtcactctgtttatggataaaagacagatggag 344bp
agaggATAACTTCGTATAGCATACATTATACGAAGTTATGTCGACagttggaccaaat
gtggataggctacaaaatgaagggtcaatgagaagtgagaactcatgggggaggttatga
ttagtgaggaagtcagaccgagaatgatggggaaagggg
```

Targeted mouse *Klk6* gene sequence after CRE excision and genotyping primers

```
ttcagaagggggcagacctgagcatccatcattctgtgaacagttctcttcagccaggt
atccataggttccgggaccctgaaggtatctcaagcccttaaaaagtaatgcaacattt
gcgtataacctatggatatctttgcatgaactctaagttatatctctagagcacctgtca
acgcagggggaacaattgctctgggtaccacctccag

CCGCGGTGGCGGCCGCTCTAGAACTAGTGGATCCCCCGGGAAGTTCCTATACTTTCT
AGAGAATAGGAACTTCGGAATAGGAACTTCGTCGATCGACCTCGAATAACTTCGTAT
AGCATACATTATACGAAGTTATGTCGAC

agttggaccaaatgtggataggctacaaaatgaagggtcaatgagaagtgagaactcatg
gggaggttatgattagtgaggaagtcagaccgagaatgatggggaaaggggccatttgggag
acagtgacaaggaaagcgatcctaagcatgaaagagaaacaaaatggga
```

Legend:
KLK mouse gene codons – in blue
KLK mouse gene introns- in black
Plasmid vector sequence – in green
Genotyping primers for the floxed allele – in fluorescent green
Genotyping primers for after CRE excision of Exon 5 – in purple
FRT site – in brown
loxP site – in orange

The *Klk6* conditional knockout strain was backcrossed to C57BL6 background for 6 generations before being utilized for experiments

The following primers were used to evaluate the status of the targeted *Klk6* sequence in the intestinal tract of mice after neo sequence removal and Cre-recombinase-mediated excision: KLK6afterCre.Forward **GGGCAGACCTGAGCATCC**, KLK6afterCre.Reverse **CATGAGTTCTCACTTCTCATTGACC**, producing 410bp PCR product; or KLK6afterCre.wt.Forward **CTTGAAGAATGACTGCTCTGAGG**, producing 316bp PCR product.

Section: KLK6 human -mouse protein alignment: the top is the human protein and the bottom the mouse. The positions of catalytic triad, histidine, aspartic acid and serine, are highlighted in yellow.

```
Query  1    MKKLMVVLSLIAAAWAEEQNKLVHGGPCDKTSHPYQAALYTSGHLLCGGVLIHPLWVLTA  60
            MK L + L L  +AW+EEQ K+VHGGPC K SHP+QAALYTSGHLLCGGVLI P WVLTA
Sbjct  8    MKMLALCLVLAKSAWSEEQEKVVHGGPCLKDSHPFQAALYTSGHLLCGGVLIDPQWVLTA  67

Query  61   AHCKKPNLQVFLGKHNLRQRESSQEQSSVVRAVIHPDYDAASHDQDIMLLRLARPAKLSE  120
            AHCKKPNLQV LGKHNLRQ E+ Q Q SV R ++HP Y+  +HD DIM++ L  P K S+
Sbjct  68   AHCKKPNLQVILGKHNLRQTETFQRQISVDRTIVHPRYNPETHDNDIMMVHLKNPVKFSK  127

Query  121  LIQPLPLERDCSANTTSCHILGWGKTADGDFPDTIQCAYIHLVSREECEHAYPGQITQNM  180
             IQPLPL+ DCS    +C ILGWGK  +GDFPDTIQCA +HLV RE+CE AYPG+ITQ+M
Sbjct  128  KIQPLPLKNDCSEENPNCQILGWGKMENGDFPDTIQCADVHLVPREQCERAYPGKITQSM  187

Query  181  LCAGDEKYGKDSCQGDSGGPLVCGDHLRGLVSWGNIPCGSKEKPGVYTNVCRYTNWIQKT  240
            +CAGD K G DSCQGDSGGPLVCG  LRGLVSWG++PCGSKEKPGVYT+VC +  WIQ
Sbjct  188  VCAGDMKEGNDSCQGDSGGPLVCGGRLRGLVSWGDMPCGSKEKPGVYTDVCTHIRWIQNI  247

Query  241  IQAK  244
            ++ K
Sbjct  248  LRNK  251
```

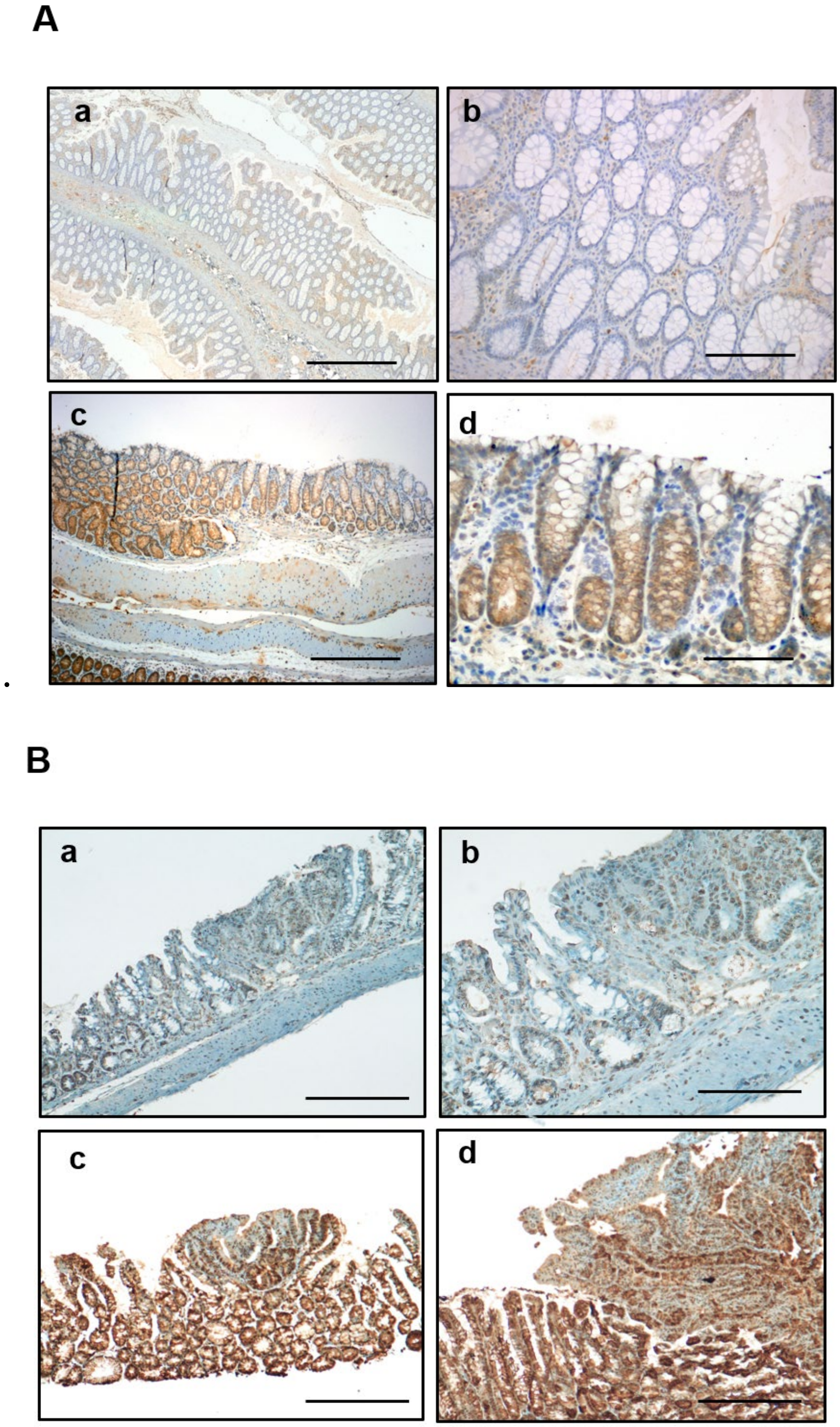

Figure S1. Validation of the specificity of a human-specific KLK6 antibody in the mouse tissue.

A. Staining of the microscopically normal mouse colon tissue of the *Apc*$^{Min/+}$ mouse with normal goat IgG control antibody (R&D Systems, Minneapolis, MN, Cat#AB-108-C, dilution 1:150) (images **a** and **b**) and a human-specific KLK6 antibody (R&D, AF2008, dilution 1:150) (images **c** and **d**). Images **a, c-** Scale bar 1 mm**; images b, d-** Scale bar 200 μm

B. Staining of the colon adenoma tissue samples from the 15-week old *Apc*$^{Min/+}$ mouse with normal goat IgG control antibody (R&D Systems, Minneapolis, MN, Cat#AB-108-C, dilution 1:150) (images **a**, and **b**). Staining of the colon adenoma tissue samples from the 15-week old *Apc*$^{Min/+}$ mouse with a human-specific KLK6 antibody (R&D, AF2008, dilution 1:150) (images **c**. and **d**). Images **, a, c-** Scale bar 1 mm**,** and images **b, d-**Scale bar 200 μm**.**

Table S1. List of antibodies used in Western blotting.

**Antibody**	**Manufacturer (City, State)**	**Primary Antibody , Dilution Factor**	**Secondary Species & Dilution Factor Cell Signaling ab (except as specified)**	**Primary Antibody Dilution Media**
Anti-β-actin	Sigma-Aldrich (St. Louis, MO)	#A5441 Clone AC-15 1:5000	Anti-mouse IgG #7076 1:10,000	5% BSA Shaking 1h at RT
E-cadherin	Cell Signaling	#3195 1:1000	Anti-rabbit IgG #7074 1:2000	5% Blotto A at $4^{O}C$ O/N shaking
β-catenin	Cell Signaling	#9562 1:1000	Anti-rabbit IgG #7074 1:2000	5% Blotto A at $4^{O}C$ O/N shaking
c-MYC	Santa Cruz Biotechnology	Sc-764 1:400	Anti-rabbit IgG #7074 1:2000	5% Blotto A at $4^{O}C$ O/N shaking
SMAD 2/3	Cell Signaling	#8685 1:1000	Anti-rabbit IgG #7074 IgG 1:2000	5% Blotto A at $4^{O}C$ O/N shaking
p-SMAD 2	Cell Signaling	#3108S 1:1000	Anti-rabbit IgG #7074 IgG 1:2000	5% Blotto A at $4^{O}C$ O/N shaking
TGF-β2	R&D	AB-12 1:200	Anti-rabbit IgG #7074	5% Blotto A at $4^{O}C$ O/N shaking
Phospo-p44/42 (phospho-Erk1/2)	Cell Signaling	#4370	Anti-rabbit IgG #7074	5% Blotto A at $4^{O}C$ O/N shaking
p44/42 (Erk1/2)	Cell Signaling	#9102	Anti-rabbit IgG #7074 1:2000	5% Blotto A at $4^{O}C$ O/N shaking
Phospho-GSK3α/β (Ser21/9)	Cell Signaling	#9331	Anti-rabbit IgG #7074 1:2000	5% Blotto A at $4^{O}C$ O/N shaking
GSK3β (S21/9)	Cell Signaling	#9315	Anti-rabbit IgG #7074 1:2000	5% Blotto A at $4^{O}C$ O/N shaking
NF-κB p65	Cell Signaling	#8242	Anti-rabbit IgG #7074 1:2000	5% Blotto A at $4^{O}C$ O/N shaking

Results

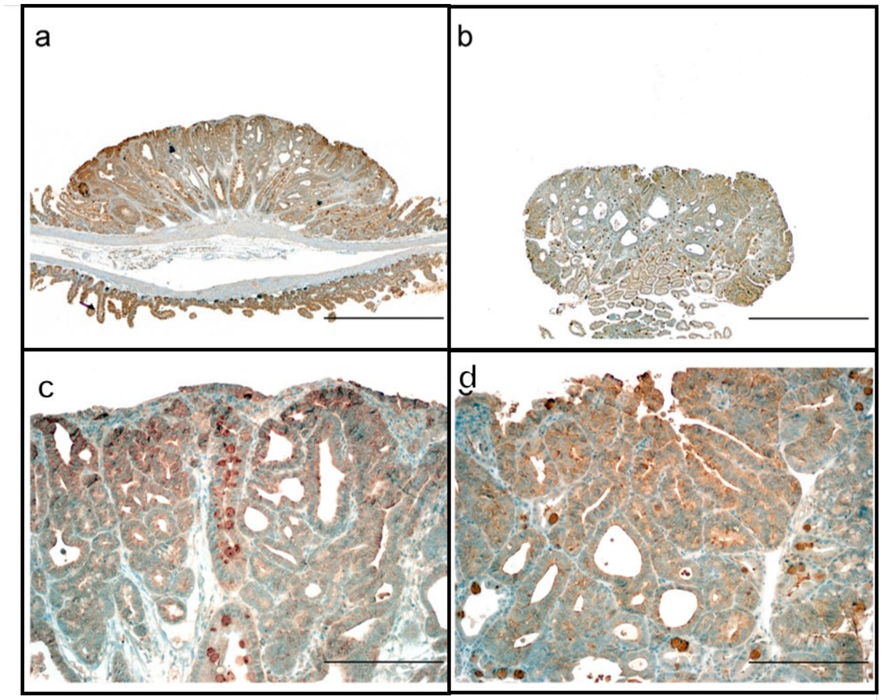

Figure S2. Representative images of KLK6 IHC staining with human-specific KLK6 antibody (R&D, AF2008, dilution 1:150) in the colon adenomas of 15 week-old *Apc*$^{Min/+}$ mice (Images **a** and **d**) and 6-month-old *CPC;Apc*$^{fl/fl}$ *;Klk6*$^{+/+}$ mice (Images **b** and **e**).. Images **a,b** - Scale bar 1 mm; images **c,d** - Scale bar 200 μm.

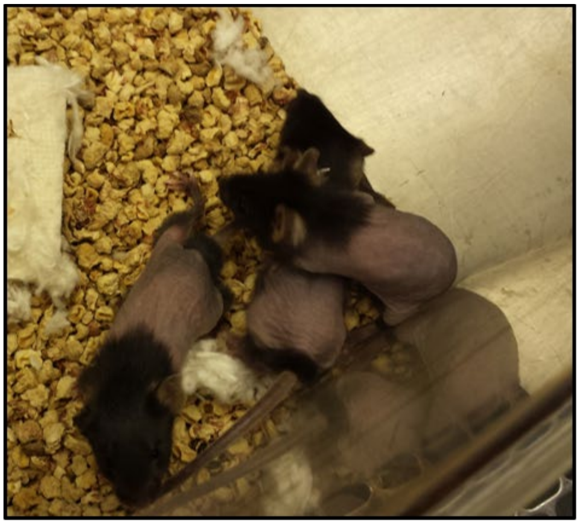

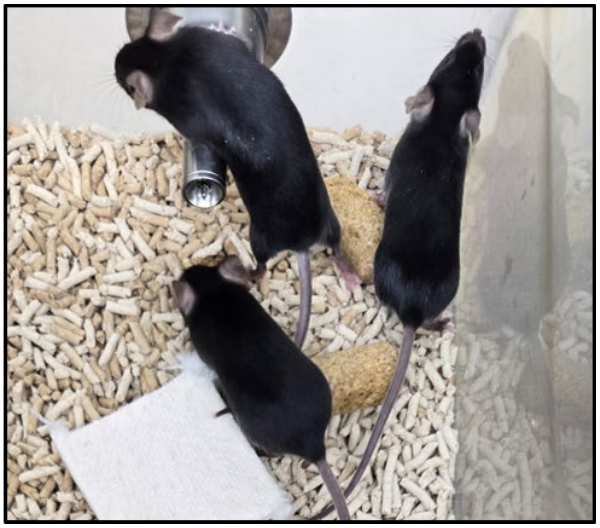

Figure S3. Example of a delay in fur growth in the 27-days old C57BL6 *Klk6*$^{loxP/loxP}$ mice (left photo) compared to the wild type C57BL6 mice of matching age (right photo). The *Klk6*$^{loxP/loxP}$ mice developed approximately 30%-40% of their fur as compared to the wild-type *Klk6* mice by visual inspection.

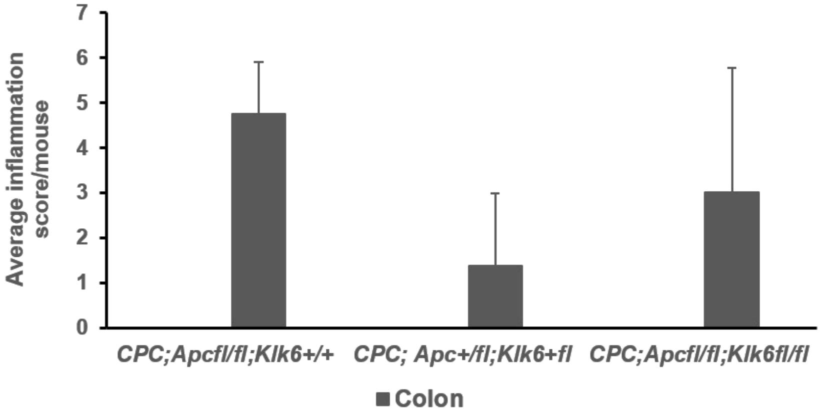

Figure S4. Average inflammation scores in the colon of animals with the homozygous inactivation of the *Apc* gene (*CPC;Apc*$^{fl/fl}$*; Klk6*$^{+/+}$), the hemizygous *Apc* and *Klk6* (*CPC;Apc*$^{+/fl}$*;Klk6*$^{+/fl}$), and the homozygous disruption of *Apc* and *Klk6* alleles (*CPC;Apc*$^{fl/fl}$*; Klk6*$^{fl/fl}$).

A

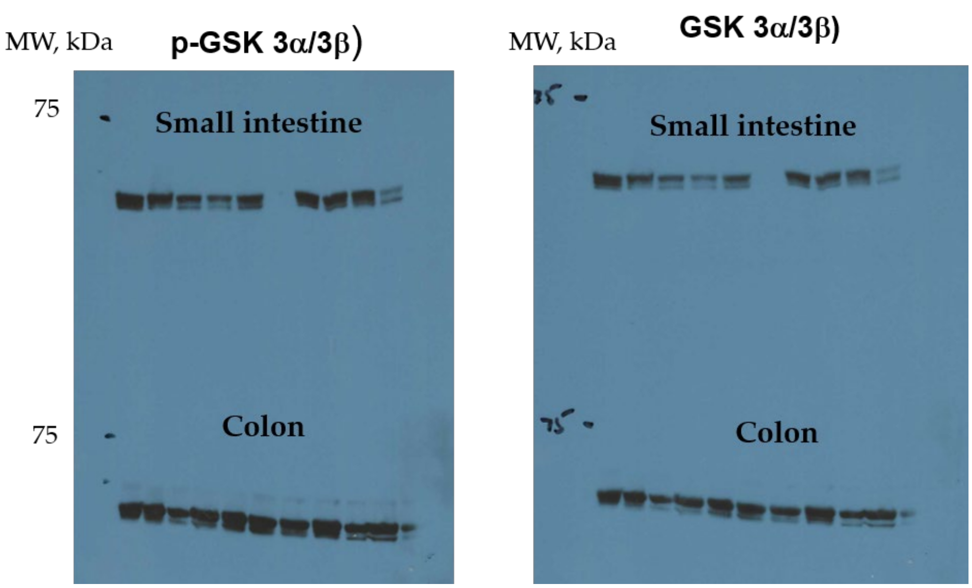

B

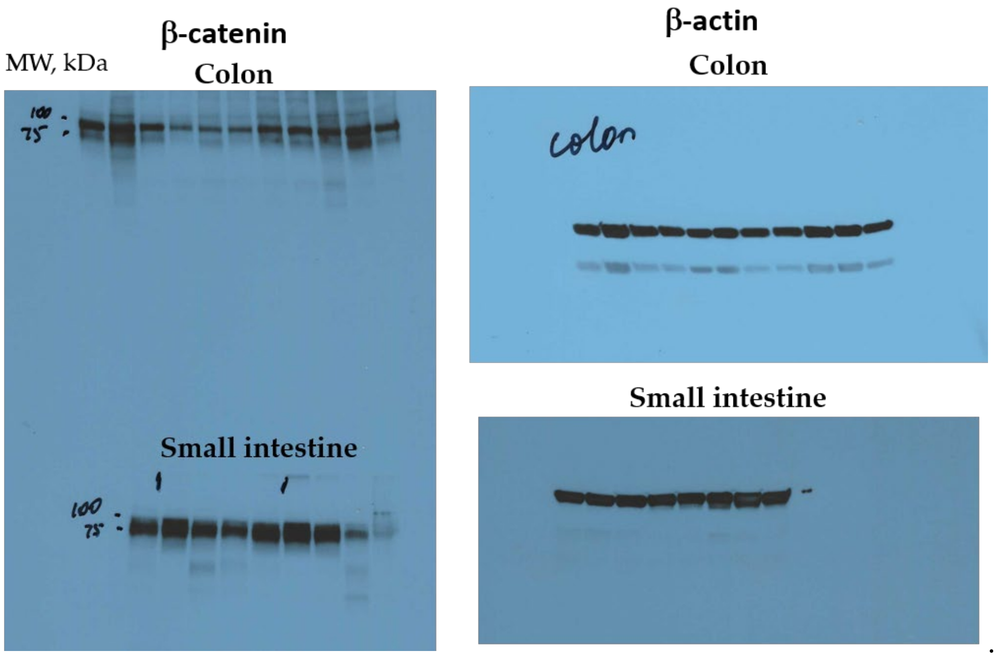

Figure S5. A. The uncropped Western blot images of p-GSK3α/3β and GSK3β proteins presented in **Figure 6A**. Molecular weight (MW) markers are shown on the left side of the images. **B.** The

uncropped Western blot images of β-catenin and β-actin proteins presented in **Figure 6C**. Molecular weight (MW) markers are shown on the β-catenin image.

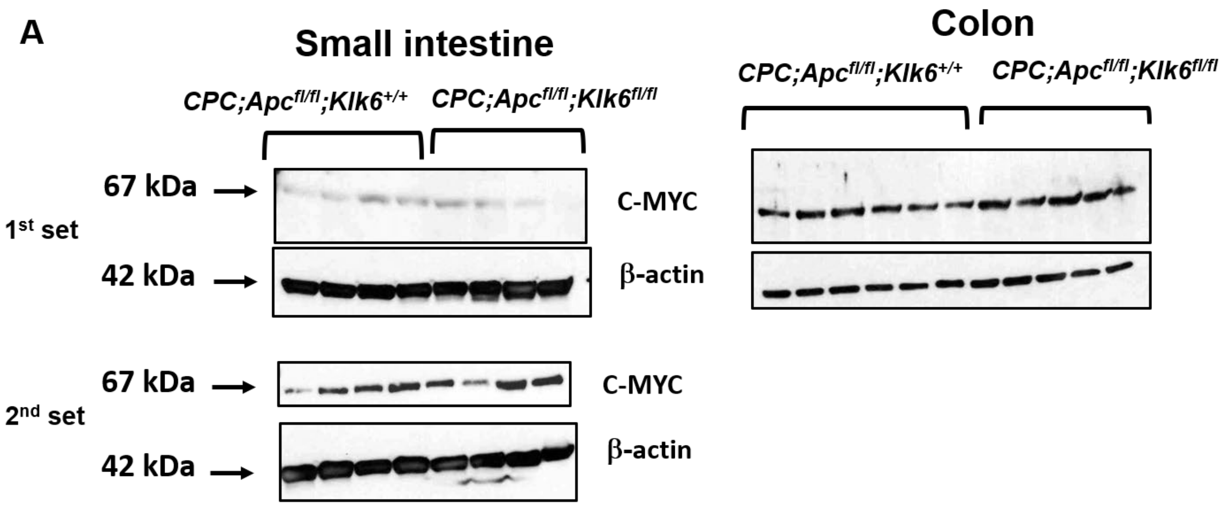

B

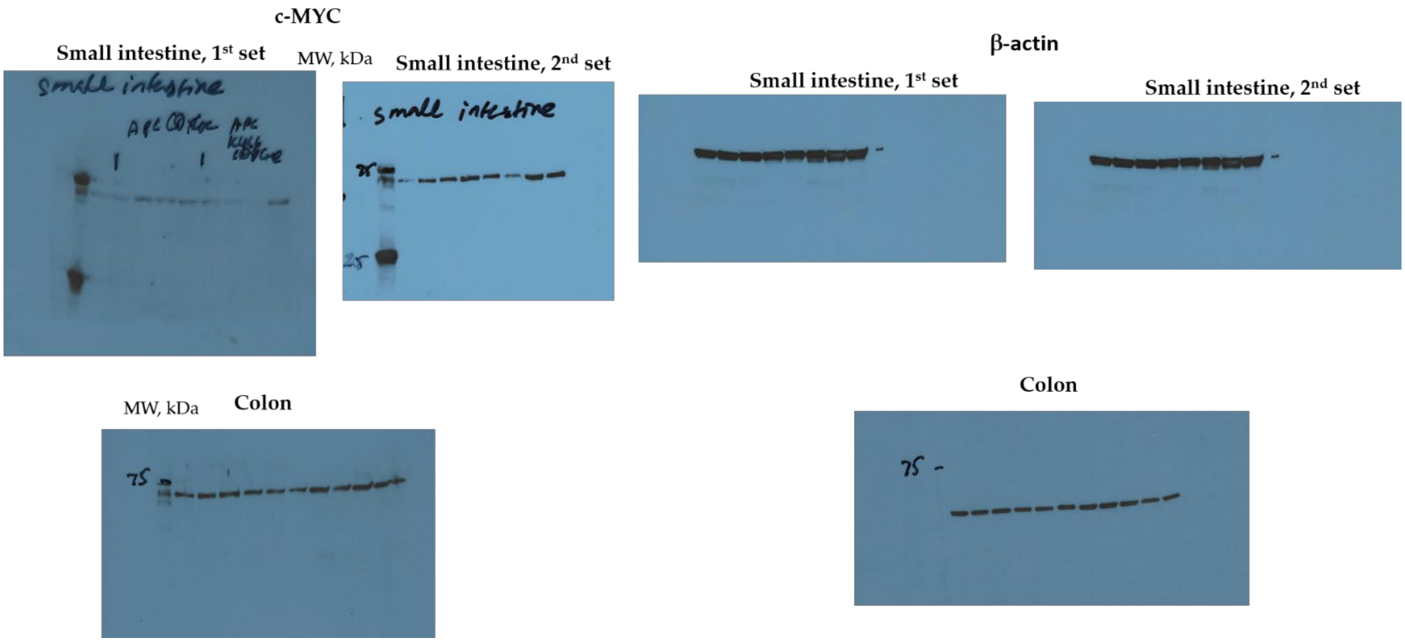

C

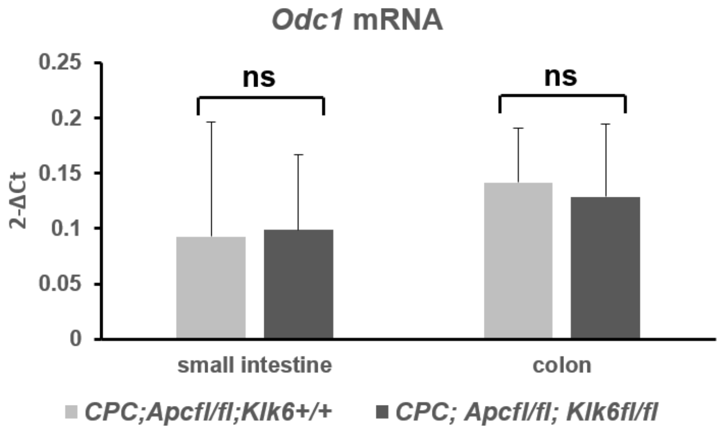

Figure S6. Evaluation of c-MYC and ODC expression in *CPC;Apc*$^{fl/fl}$ mice with the different status of *Klk6* gene. **A.** Western blot analysis of c-MYC protein levels in the small intestine (n=8 per each genotype) and in the colon of *CPC;Apc*$^{fl/fl}$;*Klk6*$^{+/+}$ (n=6); *CPC;Apc*$^{fl/fl}$;*Klk6*$^{fl/fl}$ (n=5). Each lane represents a different animal of the *CPC;Apc*$^{fl/fl}$;*Klk6*$^{+/+}$ and *CPC;Apc*$^{fl/fl}$;*Klk6*$^{fl/fl}$ genotypes. β–actin was used as a loading control. The small intestinal samples of the *CPC;Apc*$^{fl/fl}$ animals with the wild type and inactivated *Klk6* gene were run on two different gels (4 animals per genotype) and are shown here as 1st set and 2nd set. **B.** The uncropped Western blot images of c-MYC and β-actin proteins presented in **Figure S6A**. Molecular weight (MW) markers are shown on the c-MYC images. **C.** ODC mRNA levels in *CPC;Apc*$^{fl/fl}$;*Klk6*$^{+/+}$ (n=7) and *CPC;Apc*$^{fl/fl}$;*Klk6*$^{fl/fl}$ (n=8) mice measured by qPCR. Data were analysed by ANOVA non-parametric Kruskal-Wallis test (ns-p value not significant)

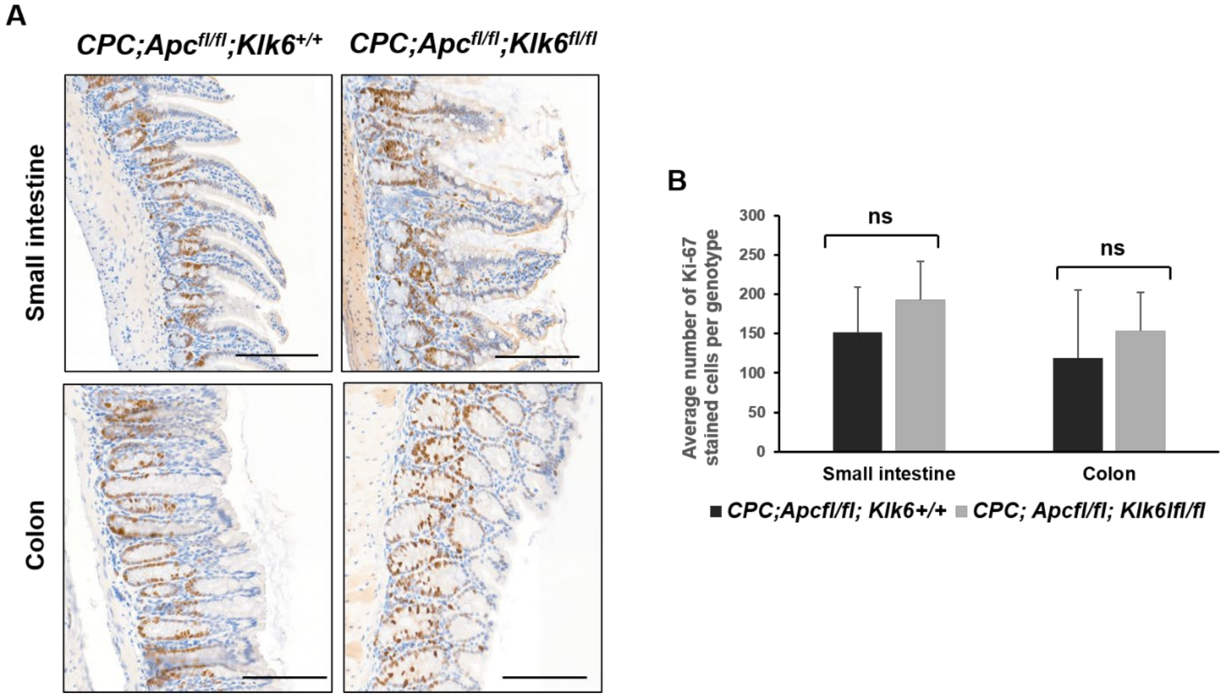

Figure S7. Ki-67 protein expression in the small intestine and colon of *CPC;Apc*$^{fl/fl}$ and *CPC;Apc*$^{fl/fl}$*;Klk6*$^{fl/fl}$ mice was analyzed by IHC. **A**. Images of Ki-67 IHC staining in the small intestine and colon of mice. Data are representative of two experiments. **B**. Quantitative analysis of Ki-67 stained cells in mice of two genotypes (*CPC;Apc*$^{fl/fl}$, n=2, and *CPC;Apc*$^{fl/fl}$*;Klk6*$^{fl/fl}$, n=4). (Scale bar 200 μm). Data were analyzed by ANOVA non-parametric Kruskal-Wallis test (ns-p value not significant).

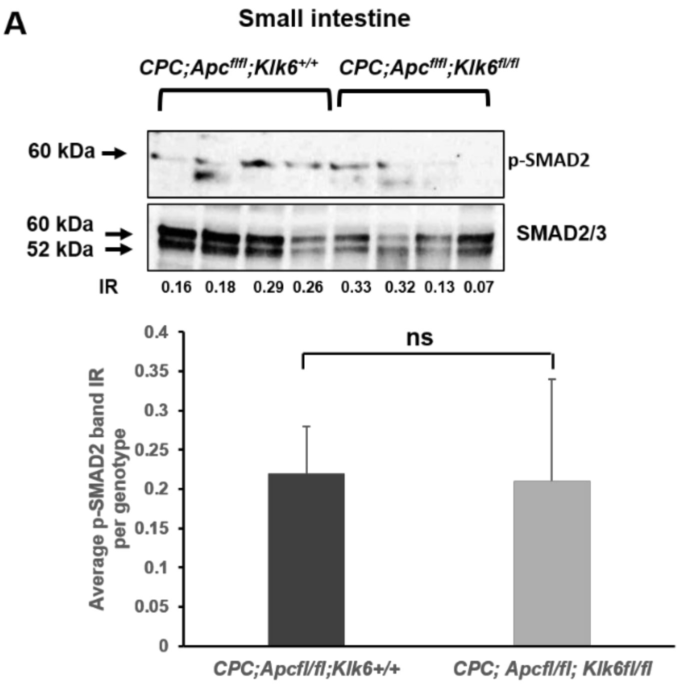
A
Small intestine
CPC;Apcflfl;Klk6+/+
CPC;Apcflfl;Klk6fl/fl
60 kDa
p-SMAD2
60 kDa
52 kDa
SMAD2/3
IR 0.16 0.18 0.29 0.26 0.33 0.32 0.13 0.07
ns
Average p-SMAD2 band IR per genotype
0.4
0.35
0.3
0.25
0.2
0.15
0.1
0.05
0
CPC;Apcfl/fl;Klk6+/+
CPC; Apcfl/fl; Klk6fl/fl

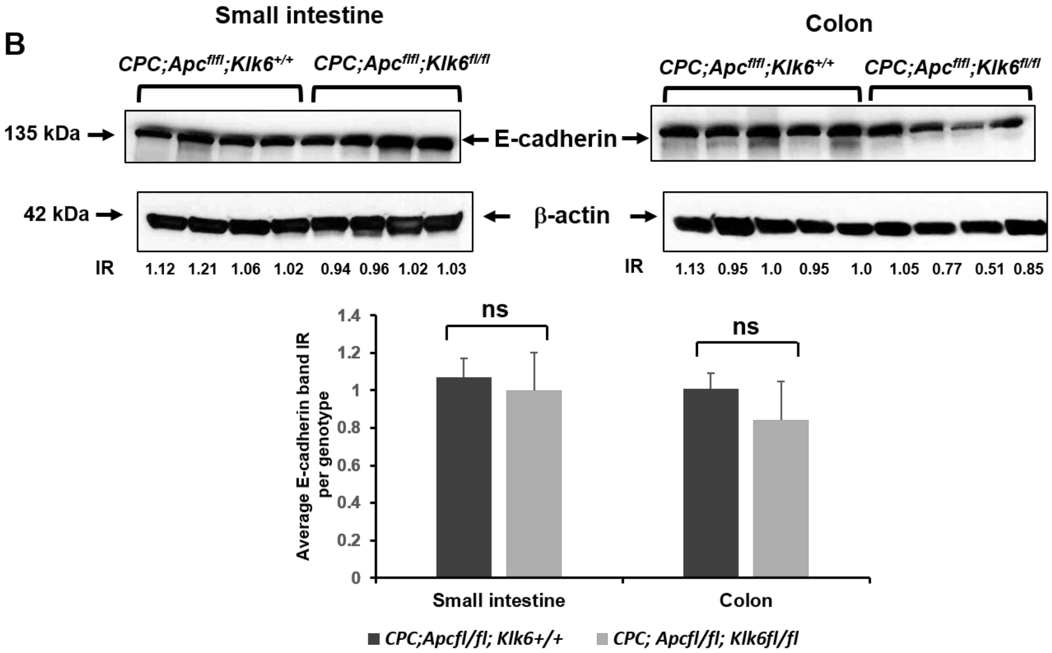
B
Small intestine
Colon
CPC;Apcflfl;Klk6+/+
CPC;Apcflfl;Klk6fl/fl
135 kDa
E-cadherin
42 kDa
β-actin
IR 1.12 1.21 1.06 1.02 0.94 0.96 1.02 1.03
IR 1.13 0.95 1.0 0.95 1.0 1.05 0.77 0.51 0.85
ns
ns
Average E-cadherin band IR per genotype
1.4
1.2
1
0.8
0.6
0.4
0.2
0
Small intestine
Colon
CPC;Apcfl/fl; Klk6+/+
CPC; Apcfl/fl; Klk6fl/fl

C.

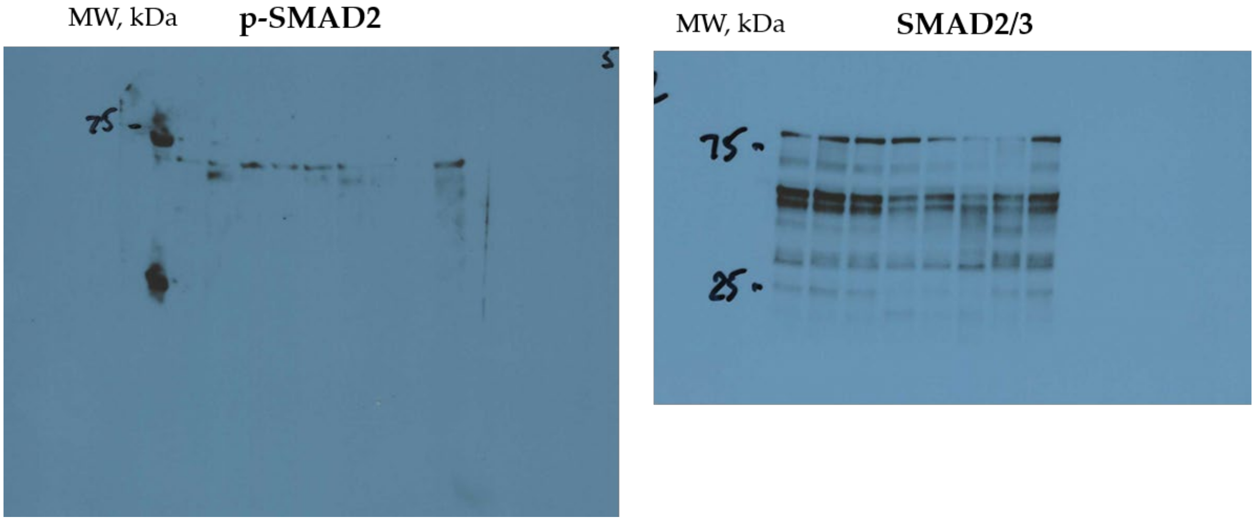

D.

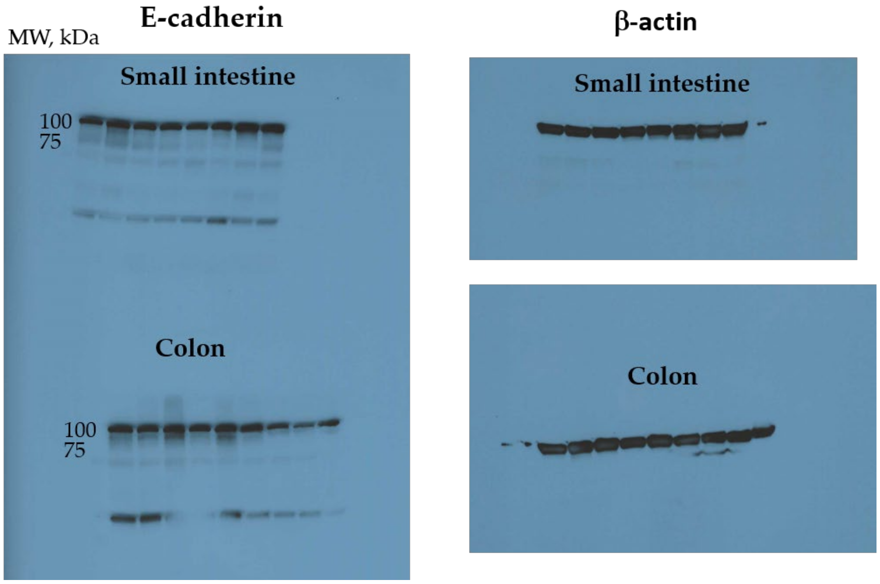

Figure S8. SMAD signaling is not altered in *CPC;Apc*$^{fl/fl}$ mice upon disruption of *Klk6* gene. A. Expression of phosphorylated SMAD2 (p-SMAD2) and SMAD2/3 proteins in the small intestine of *CPC;Apc*$^{fl/fl}$ *Klk6*$^{+/+}$ mice and *CPC;Apc*$^{fl/fl}$*;Klk6*$^{fl/fl}$ mice (n=4 mice per genotype) measured by Western blotting. The intensities of the p-SMAD and SMAD2/3 bands in the small intestine of each animal were quantified using ImageJ. The intensity ratios (IR) of p-SMAD2 to SMAD2/3

bands for each lane are included. Graph represents the average normalized p-SMAD2 protein level in mice of *CPC;Apc*$^{fl/fl}$*;Klk6*$^{+/+}$ and *CPC;Apc*$^{fl/fl}$*;Klk6*$^{fl/fl}$ genotypes. Data were analyzed using ANOVA single factor test (ns-p value not significant). The figure is representative of two independent experiments. **B**. Western blot analysis of E-cadherin protein level in the small intestine and colon of *CPC;Apc*$^{fl/fl}$ *Klk6*$^{+/+}$ mice (n=4, small intestine, n=5 colon) and *CPC;Apc*$^{fl/fl}$*;Klk6*$^{fl/fl}$ mice (n=4). The β-actin protein Western blotting was used as a loading control. The intensities of the E-cadherin and β-actin bands in the small intestine and colon of each animal were quantified using ImageJ. The intensity ratios (IR) of E-cadherin to β-actin bands for each lane are included. The graph represents the average normalized E-cadherin protein level in *CPC;Apc*$^{fl/fl}$*;Klk6*$^{+/+}$ and *CPC;Apc*$^{fl/fl}$*;Klk6*$^{fl/fl}$ mice. Data were analyzed using ANOVA single factor test (ns-p value not significant). The figure is representative of two independent experiments. **C**. The uncropped Western blot images of p-SMAD2 and SMAD2/3 proteins presented in **Figure S8A**. Molecular weight (MW) markers are shown on the left side of the images. **D**. The uncropped Western blot images of E-cadherin and β-actin proteins presented in **Figure S8B**. Molecular weight (MW) markers are shown on the E-cadherin image.

A.

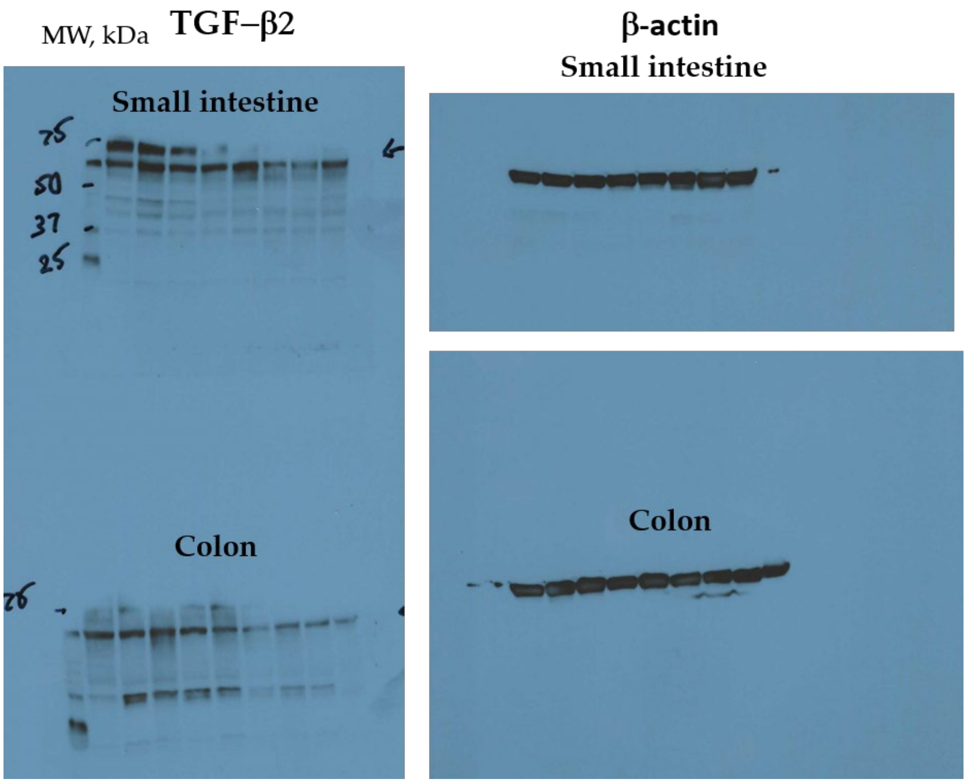

MW, kDa
TGF–β2
Small intestine
75
50
37
25
Colon
β-actin
Small intestine
Colon

B.

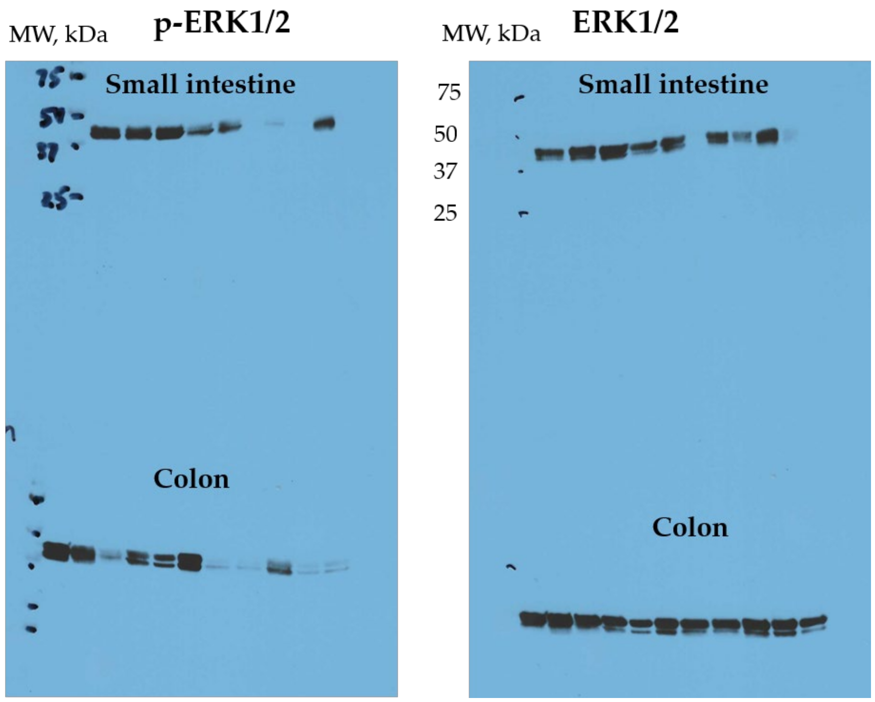

MW, kDa
p-ERK1/2
Small intestine
Colon
MW, kDa
ERK1/2
Small intestine
75
50
37
25
Colon

Figure S9. A. The uncropped Western blot images of TGF-β2 and β-actin proteins presented in **Figure 7A**. Molecular weight (MW) markers are shown on the TGF-β2 image.

B. The uncropped Western blot images of p-ERK1/2 and ERK1/2 proteins presented in **Figure 7C**. Molecular weight (MW) markers are shown on the left of the images.
